# Functionalizing Collagen Membranes with MSC-Conditioned Media Promotes Guided Bone Regeneration in Rat Calvarial Defects

**DOI:** 10.3390/cells12050767

**Published:** 2023-02-28

**Authors:** Siddharth Shanbhag, Carina Kampleitner, Niyaz Al-Sharabi, Samih Mohamed-Ahmed, Karol Ali Apaza Alccayhuaman, Patrick Heimel, Stefan Tangl, Andreas Beinlich, Neha Rana, Mariano Sanz, Einar K. Kristoffersen, Kamal Mustafa, Reinhard Gruber

**Affiliations:** 1 Department of Immunology and Transfusion Medicine, Haukeland University Hospital, 5021 Bergen, Norway; 2 Center for Translational Oral Research (TOR), Department of Clinical Dentistry, Faculty of Medicine, University of Bergen, 5009 Bergen, Norway; 3 Karl Donath Laboratory for Hard Tissue and Biomaterial Research, University Clinic of Dentistry, Medical University of Vienna, 1090 Vienna, Austria; 4 Ludwig Boltzmann Institute for Traumatology, The Research Center in Cooperation with AUVA, 1200 Vienna, Austria; 5 Austrian Cluster for Tissue Regeneration, 1200 Vienna, Austria; 6 Department of Oral Biology, University Clinic of Dentistry, Medical University of Vienna, 1090 Vienna, Austria; 7 Department of Earth Science, Faculty of Mathematics and Natural Sciences, University of Bergen, 5009 Bergen, Norway; 8 ETEP Research Group, Faculty of Odontology, University Complutense of Madrid, 28040 Madrid, Spain; 9 Department of Periodontology, School of Dental Medicine, University of Bern, 3010 Bern, Switzerland

**Keywords:** mesenchymal stromal cells, conditioned media, guided bone regeneration, bone tissue engineering, regenerative medicine

## Abstract

Functionalizing biomaterials with conditioned media (CM) from mesenchymal stromal cells (MSC) is a promising strategy for enhancing the outcomes of guided bone regeneration (GBR). This study aimed to evaluate the bone regenerative potential of collagen membranes (MEM) functionalized with CM from human bone marrow MSC (MEM-CM) in critical size rat calvarial defects. MEM-CM prepared via soaking (CM-SOAK) or soaking followed by lyophilization (CM-LYO) were applied to critical size rat calvarial defects. Control treatments included native MEM, MEM with rat MSC (CEL) and no treatment. New bone formation was analyzed via micro-CT (2 and 4 weeks) and histology (4 weeks). Greater radiographic new bone formation occurred at 2 weeks in the CM-LYO group vs. all other groups. After 4 weeks, only the CM-LYO group was superior to the untreated control group, whereas the CM-SOAK, CEL and native MEM groups were similar. Histologically, the regenerated tissues showed a combination of regular new bone and hybrid new bone, which formed within the membrane compartment and was characterized by the incorporation of mineralized MEM fibers. Areas of new bone formation and MEM mineralization were greatest in the CM-LYO group. Proteomic analysis of lyophilized CM revealed the enrichment of several proteins and biological processes related to bone formation. In summary, lyophilized MEM-CM enhanced new bone formation in rat calvarial defects, thus representing a novel ‘off-the-shelf’ strategy for GBR.

## 1. Introduction

The reconstruction of complex bone defects, where tissue deficiency occurs tri-dimensionally, is a clinical challenge [1]. In the alveolar bone, the recommended treatment approaches for such defects have been either guided bone regeneration (GBR) using autologous bone grafts in combination with a bone substitute material and a barrier membrane or autogenous or allogeneic bone blocks [2,3]. Although GBR has demonstrated a high degree of clinical success and predictability [4,5], in the presence of large defects, the need for extensive autologous bone harvesting may result in additional patient morbidity and risks of clinical complications [6].

Bone tissue engineering is increasingly being used to overcome these limitations [7] by combining autologous transplantation of ex vivo expanded adult mesenchymal stromal cells (MSC), usually from the bone marrow (BMSC), with biomaterial scaffolds [8,9]. However, this approach has important logistic and regulatory complications that limit its efficiency and predictability. In fact, in a recent meta-analysis, our group reported that the clinical evidence for effectiveness of cell therapy was limited, i.e., there were relatively small effect sizes vs. traditional GBR/grafting procedures, and these were mainly limited to studies of maxillary sinus augmentation [7]. Moreover, the large-scale translation of this strategy is limited by the need for expensive Good Manufacturing Practice (GMP)-grade laboratories for ex vivo cell expansion and stringent regulation of MSC as Advanced Therapeutic Medicinal Products (ATMP) by health authorities. Furthermore, the traditional hypothesis that MSC act via engraftment, differentiation and replacement at the injury site has, in recent years, been challenged by evidence of a predominantly paracrine mechanism of action [10,11].

It is widely accepted that MSC may exert their beneficial effects by secreting a wide range of bioactive factors, including soluble proteins (growth factors, cytokines and chemokines), lipids, nucleic acids and extracellular vesicles at or near the site of injury [11,12,13]. These factors, in turn, stimulate tissue-resident progenitor (osteogenesis), endothelial (angiogenesis) and immune cells (immune modulation) to drive the subsequent regeneration processes [14]. These findings have provided the biological basis for developing ‘cell-free’ strategies, which use the secretome contained in MSC-conditioned media (CM) to stimulate tissue regeneration. An additional advantage of this strategy is the possibility of storing and using MSC-CM as ‘off-the-shelf’ products [15]. Although the preclinical efficacy of MSC-CM for bone regeneration has previously been reported [16,17], data for the optimal dose(s) and mode(s) of CM delivery are lacking. 

GBR techniques are based on the use of barrier membranes that act as occlusive barriers to the rapidly proliferating cells of epithelial and connective tissues, while promoting repopulation with slower-growing osteoprogenitor cells [18,19]. Bioabsorbable collagen membranes (MEM) are the most frequently used membranes in GBR, and are either applied alone or combined with bone substitute materials [20]. In addition to functioning as occlusive barriers, MEM have also shown an inherent biological activity via their ability to adsorb and release signaling molecules, e.g., growth factors [21,22]. This property has been exploited in several preclinical studies where MEM have been functionalized with bioactive molecules, e.g., bone-derived proteins [23,24] and recombinant growth factors (see review [4]). We have previously demonstrated that MEM can adsorb the growth factor-activity from human biological products ex vivo [25]. Thus, it is reasonable to hypothesize that MEM can also adsorb bioactive factors from CM and serve as carriers or ‘scaffolds’ in GBR settings [14,25]. To test this hypothesis, we propose using the calvarial critical size defect model in rodents, since this model is extensively used for testing other GBR strategies [26]. Thus, the objective of the present study was to investigate the efficacy of CM-functionalized MEM (MEM-CM) for promoting GBR in vivo in rat calvarial defects. A secondary objective was to compare two different methods, i.e., soaking vs. lyophilization of CM, for MEM functionalization.

## 2. Methods

### 2.1. Cell Culture

The use of human cells and tissues was approved by the Regional Committees for Medical Research Ethics in Norway (2013-1248/REK-sør-øst and 2016-1266/REK-nord). Bone marrow specimens were obtained following parental consent from five independent donors (2 females and 3 males; 8–10 years) undergoing reconstructive surgery at the Department of Plastic Surgery, Haukeland University Hospital. BMSC were isolated and expanded following previous protocols [27]. Briefly, the cells were cultured in T75 or T175 flasks (Thermo Fisher Scientific, Carlsbad, CA, USA) using sterile filtered growth media (GM) comprising of Dulbecco’s Modified Eagle’s medium (DMEM, Invitrogen, Carlsbad, CA, USA) supplemented with 5% (*v/v*) pooled human platelet lysate (HPL; Bergenlys, Bergen, Norway), 1% (*v/v*) penicillin/streptomycin (GE Healthcare, South Logan, UT, USA) and 1 IU/mL heparin (Leo Pharma AS, Lysaker, Norway) [27]. The cells were sub-cultured and expanded under standard incubation conditions, i.e., 37 °C and 5% CO_2_, following a validated protocol with a seeding density of 4000 cells/cm^2^ [28]. Passage 1 (p1) and 2 (p2) BMSC were characterized based on immunophenotype and multi-lineage differentiation potential, as previously reported [27]. For all the cell cultures, growth and morphology were monitored regularly under an inverted light microscope (Nikon Eclipse TS100, Tokyo, Japan).

### 2.2. CM Preparation

Pooled CM were prepared from BMSC (n = 3 donors) as previously described [29]. Briefly, p1 and p2 BMSC were expanded in T175 flasks in GM until 70–80% confluency under standard incubation. At this point, the cells were washed three times with phosphate-buffered saline (PBS; Invitrogen), and then cultured in plain DMEM (without HPL or antibiotics) for another 48 h to produce CM. After 48 h, the CM from p1 and p2 BMSC from each of the three donors were collected, pooled, centrifuged at 4000× *g* for 10 min to remove any debris, aliquoted and stored at −80 °C. The CM were further concentrated using 3 kDa Amicon Ultra-15 centrifugal filter devices (Merck Millipore, Billerica, MA, USA) using the manufacturers protocol. Briefly, following the equilibration of filter devices with PBS, the CM were centrifuged at 4000× *g* for 30 min at 4 °C, followed by PBS buffer exchange and another centrifugation cycle (4000× *g* for 30 min), resulting in concentrated CM (~30-fold). Based on previous reports [15,30], mannitol (Sigma Aldrich, St. Louis, MO, USA) was added as a cryo-preservative (0.5% *v/v*), and the concentrated CM were then used for MEM functionalization. 

### 2.3. MEM Functionalization and Bioassay

Bi-layered, non-cross-linked MEM (25 mm × 25 mm; Bio-Gide^®^, Geistlich Pharma, Wolhusen, Switzerland) were used in this study. The MEM were cut using sterile scissors into smaller pieces (7 mm × 6 mm) and incubated with 100 μL of serum-free DMEM (control) or concentrated CM at 37 °C for 1 h based on previous experiments where the incubation conditions for optimal adsorption of proteins were determined [22]. For equal comparison, mannitol was also added to serum-free DMEM at a final concentration of 0.5% (*v/v*). After 1 h, the supernatants were aspirated, and MEM soaked with serum-free DMEM (native MEM, control group) and half of the MEM soaked with CM (CM-SOAK) were stored at 4 °C. The remaining MEM soaked with CM were stored in a −80 °C freezer for subsequent lyophilization. Lyophilization of the MEM-CM was performed in a FreeZone™ freeze dryer (Labconco, Kansas, MO, USA) at 0.014 mBar of pressure and at −51 °C. The lyophilized MEM-CM (CM-LYO) were stored at 4 °C until their use in the experiments (up to 24 h). As a bioassay, the effects of CM alone and MEM-CM (CM-SOAK and CM-LYO) on BMSC were tested via a quantitative real-time polymerase chain reaction (qPCR) using TaqMan^®^ real-time PCR assays (Thermo Scientific) as previously described [31]. Primary BMSC (different from those used for CM preparation) were exposed to GM and CM in a monolayer culture, and to CM-SOAK, CM-LYO and native MEM in a three-dimensional (3D) culture for 48 h. Expressions of osteogenesis-related genes (Supplementary Table S1) were assessed as previously described [31].

### 2.4. Cell Viability on MEM

To test the cytocompatibility of the functionalized MEM-CM (CM-SOAK and CM-LYO), i.e., whether the resident cells could populate the MEM following in vivo implantation, the in vitro viability of rat MSC (rMSC) seeded on native and functionalized MEM was determined after 1 and 3 days using the LIVE/DEAD cell viability assay (Invitrogen) as previously described [32]. Briefly, previously isolated rMSC [33] were cultured in DMEM supplemented with 10% (*v/v*) fetal bovine serum (FBS) and 1% (*v/v*) penicillin/streptomycin; p3-5 cells were used in experiments. Pooled rMSC (~10^5^ cells) were seeded on MEM and allowed to attach for 1 h before supplementing them with the corresponding growth media for 1–3 days. At each time point, MEM were stained and observed under a confocal microscope (Andor Dragonfly 5050, Oxford Instruments, Abingdon, UK) coupled with Imaris software ver. 9.5.1 (Oxford Instruments), and green (live) and red (dead) cells were visualized qualitatively in each condition. For the animal experiment, pooled rMSC (1.5 × 10^6^ cells) were seeded on the MEM as described above and cultured in growth media for 24 h before implantation. Cell viability was confirmed just prior to implantation using the aforementioned viability assay.

### 2.5. Rat Calvaria Defect Model

The calvarial defect model in rats was performed following ethical approval (Norwegian Animal Research Authority, FOTS-17443) and in accordance with the ARRIVE guidelines, as previously described [32]. Briefly, 20 male Lewis rats (LEW/OrlRj, Janvier Labs, Le Genest-Saint-Isle, France) that were 8 weeks old and weighing 200–350 g were used. Following acclimatization, the animals were anesthetized (Sevoflurane, Abbott Laboratories, Berkshire, UK), and two full-thickness defects were surgically created, one in each parietal bone, using a trephine bur with an outer diameter of 5 mm (Meisinger GmbH, Neuss, Germany) under saline irrigation. Three animals died during the surgery due to anesthesia-related complications; therefore 17 animals were available for the experiment. The following treatments were then randomly applied to the defects: CM-LYO (n = 8), CM-SOAK (n = 8), MEM seeded with allogeneic pooled rMSC (CEL, 1.5 × 10^6^ cells; n = 7), native MEM soaked with serum-free DMEM (MEM; n = 6) and no treatment (‘empty’ defects; n = 5). Membranes were fixed to the calvaria using 3–5 μL of tissue adhesive (Histoacryl^®^, B. Braun, Tuttingen, Germany) at the defect edges [34,35]; the fixation of MEM is advised to prevent micromovements and promote healing [36]. Randomization of defects/groups was performed using the Research Randomizer online software [37], and the animals were coded via ear clips. For all subsequent handling/analyses, the animals/specimens were identified by numbers to facilitate blinding of the observers to the treatment groups. After 2 weeks, the animals were subjected to in vivo micro-computed tomography (μCT), and after 4 weeks, they were euthanized with an overdose of CO_2_. The primary outcome was new bone formation after 2 weeks via in vivo μCT and after 4 weeks via ex vivo μCT, histology and histomorphometry. The secondary outcomes included the characterization of new bone tissues via scanning electron microscopy (SEM), microhardness testing, relative bone density and Raman spectroscopy.

### 2.6. μ CT

To track early in vivo bone regeneration, the live animals were scanned at 2 weeks post-surgery under anesthesia using a small-animal CT scanner and Mediso workstation (both from nanoScan Mediso, Budapest, Hungary) with a voxel size of 40 μm (resolution), 70 kV energy, an exposure time of 300 ms, 720 projections and 1:1 binning. After a period of observation, the animals were returned to their original cages and housing locations until euthanasia. After 4 weeks, the calvaria were harvested and fixed in 10% buffered formalin. The specimens were scanned using a SCANCO 50 μCT scanner (SCANCO Medical AG, Bruttisellen, Switzerland) at 90 kV and 200 μA with an isotropic resolution of 20 μm. Reconstruction and analysis were performed as previously described [32]. Briefly, scans were reconstructed using Amira software (Thermo Scientific) by orienting the drill direction along the *Z*-axis, with the defect in the approximate center of the image. Using ImageJ software (NIH, Bethesda, MD, USA), a standardized volume of interest (VOI) including the entire thickness of the calvaria and excluding 0.5 mm of marginal bone was defined for each defect. Specific density thresholds were defined for in vivo and ex vivo µCT scans (based on scanning resolutions) and percentages of new bone volume relative to total defect volume (BV/TV%) and bone coverage (%) were calculated in ImageJ (NIH) using custom defined rulesets.

### 2.7. Histology and Histomorphometry

After μCT scanning, the calvaria specimens were processed for undecalcified histology as previously described [32]. Briefly, the specimens were dehydrated in ascending grades of alcohol and embedded in light-curing resin (Technovit 7200 + 1% benzoyl peroxide, Kulzer & Co., Wehrheim, Germany). The blocks were further processed using EXAKT cutting and grinding equipment (EXAKT Apparatebau, Norderstedt, Germany). Standardized thin-ground sections (~100 μm) were prepared in the centre of each defect, parallel to the sagittal suture and perpendicular to the parietal bone, and stained with Levai-Laczko dye (Morphisto GmbH, Frankfurt, Germany). In this staining process, mature bone appears light pink, woven bone is dark pink and soft tissues (including collagen) are dark blue. The sections were scanned using an Olympus BX61VS digital virtual microscopy system (DotSlide 2.4, Olympus, Tokyo, Japan) with a 20× objective, resulting in a resolution of 0.32 µm per pixel.

Histomorphometric analysis was performed to analyze the tissue components filling the defects as previously described [38]. Briefly, the scanned images were manually segmented using Photoshop CS 6 (Adobe Systems Inc., San Jose, CA, USA) and quantified using a custom script in ImageJ (NIH). Two regions of interest (ROI) were defined for each sample based on the position of the membrane in relation to the defect: the central defect region, delimited superiorly by the MEM, inferiorly by the dura and laterally by the defect edges, and the defect edge or ‘side’ region, which was the area adjacent to the central defect on both sides (Supplementary Figure S1). In both ROIs, the respective areas of new bone without embedded MEM fibers (hereafter termed ‘new bone’), new bone with embedded MEM fibers (hereafter termed ‘hybrid bone’), total new bone (sum of new and hybrid bone), mineralized MEM fibers, residual MEM (non-mineralized MEM fibers) and soft tissue were measured, and corresponding percentages were calculated as a ratio of the ROI area.

### 2.8. Characterization of New Bone Tissues

The structural, mechanical and compositional properties of new bone tissues were analyzed based on SEM, microhardness, relative bone density and Raman spectroscopy. The objective herein was to compare the different tissue types, i.e., regular new bone and hybrid new bone, and not the different treatment conditions. Representative sections from each experimental group (MEM, CM-LYO, CM-SOAK and CEL) were used, and native calvarial bone was analyzed as a control.

**SEM:** The ultrastructure of the new bone tissues, i.e., regular new bone and hybrid bone, was further analyzed using SEM. Briefly, back-scattered electron imaging of carbon coated resin-embedded calvaria sections was performed using a Zeiss Supra 55VP microscope (Carl Zeiss, Oberkochen, Germany), with an acceleration voltage of 15 kV and an 8 mm working distance.

**Microhardness:** Vickers microhardness testing was performed as previously described [39]. Briefly, micro-indentations were created on the tissue surfaces using an MHT-10 microhardness tester equipped with a Vickers diamond indenter tip and a video measuring system (Anton Paar, Graz, Austria) attached to a light microscope with a 50× objective (Leica DMR, Wetzlar, Germany). A load of 50 g was applied for 10 s to produce each indentation; at least 10 separated indentations were made per tissue type, per section. The length of the diagonals of each indentation was measured using the inbuilt software (Anton Paar), and a Vickers hardness value (H_V_) was automatically calculated.

**Relative bone density:** The selected sections were scanned using a SkyScan 1172 μCT scanner (Bruker, Kontich, Belgium) with an X-ray source of 60 kV/200 µA and 0.5 mm aluminum filter for a resolution of 13.3 µm; beam hardening was adjusted to compensate for the difference in density between the plastic microscope slides. A maximum intensity projection of each slide was created, and using a custom ImageJ script, the average intensity in each tissue type was measured. Details of the measurement protocol are presented in the Supplementary Materials.

**Raman spectroscopy:** This technique was used to study bone composition via an estimation of the crystallinity and mineral-to-matrix ratio. Raman spectra of each tissue type were collected using a confocal Raman microscope (LabRam, Horiba Jobin Yvon, Edison, NJ, USA) equipped with a 488 nm excitation laser and 50× objective coupled with the LabSpec ver. 5 software (Horiba Jobin Yvon) with the following settings: a spatial resolution of 0.5 cm^−1^, a spectral range of 400–1800 cm^−1^ and 10 accumulations with 1 s exposure time per measurement. The spectral measurements were calibrated using a silicon standard, and at least five measurements were taken per tissue type per section. The spectra were processed using a custom script in Matlab (Mathworks, Natick, MA, USA) for the selected peaks of interest, i.e., *v*_1_ phosphate (*v*_1_ PO_4_^3^) at ~960 cm^−1^, representing the mineral/inorganic phase of bone, and CH_2_ wag at ~1448 cm^−1^, representing the organic/matrix phase, i.e., collagens, lipids and non-collagenous proteins [40]. Background fluorescence correction and smoothing using the Savitzky–Golay polynomial function in the 2nd order were applied to the spectra in the appropriate wave number range (±80 cm^−1^) using a custom MatLab script. The following parameters were determined for the selected peaks of interest: peak height, peak area and the full peak width at half maximum intensity (FWHM). To ensure the correct identification of the different tissue types, stained histological sections were used, which resulted in an additional background peak from the dye (pararosaniline); the dye-peak at 913 cm^−1^ [41] could be clearly differentiated from the bone peaks and did not interfere with the analysis. The following compositional parameters were then calculated: *crystallinity*, represented by the inverse of the full peak width at half maximum intensity (FWHM^−1^) for *v*_1_ PO_4_^3^, and mineral/matrix ratio, represented by the peak height ratio of *v*_1_ PO_4_^3^ to CH_2_ wag.

### 2.9. Liquid Chromatography with Tandem Mass Spectrometry (LC-MS/MS) of Lyophilized CM

The proteomic composition of the pooled CM were analyzed using LC-MS/MS as previously described [42]. Briefly, the total protein concentration was measured using a bicinchoninic acid assay (Pierce BCA Kit, Thermo Fisher), and 10 μg of lyophilized protein was processed to obtain tryptic peptides. About 0.5 µg protein as tryptic peptides dissolved in 2% acetonitrile and 0.5% formic acid was injected into an Ultimate 3000 RSLC system connected online to a Exploris 480 mass spectrometer equipped with EASY-spray nano-electrospray ion source (all from Thermo Scientific, Sunnyvale, CA, USA). Additional details of LC-MS/MS are reported in the Supplementary Materials.

### 2.10. Bioinformatic Analysis

The LC-MS/MS raw files were searched using Proteome Discoverer software (version 2.5.0.400; Thermo Scientific). Perseus software (version 2.3.0.1; Max Planck Institute for Biochemistry, Martinsread, Germany) was used to process and filter the results. An over-representation analysis of the exclusive proteins in each CM group was performed using the WebGestalt tool (wGSEA) [43,44]. Gene ontology (GO) slim subsets were retrieved based on the human genome (Homo sapiens) as a reference. Relevant GO terms (Homo sapiens) for bone-related biological processes were retrieved from the QuickGO database (https://www.ebi.ac.uk/QuickGO/, accessed on 14 November 2022), and the corresponding gene names were compared to the proteins identified in CM [42]. A list of identified bone-related proteins is presented in Supplementary Table S2.

### 2.11. Multiplex Immunoassay

The Quantibody Human Bone Metabolism Array Q1 (RayBiotech Inc., Norcross, GA, USA) was used to analyze 31 bone related cytokines (Supplementary Table S3) according to the manufacturer’s protocol. This array is based on the sandwich enzyme-linked immunosorbent assay (ELISA) technology, and each antibody is spotted in quadruplicate. Array hybridization was performed using concentrated CM (0.1–0.2 mg/mL of total protein) and standard cytokines. Array scanning was performed using a laser scanner (GenePix 4000B, Axon Instruments, San Jose, CA, USA) at different photomultiplier tube gains. Data extraction was performed using the GenePix Pro software ver. 5.0 (Axon Instruments). Concentrations of candidate proteins were calculated based on linear standard curves and normalized to the corresponding total protein concentration.

### 2.12. Statistical Analysis

Statistical analysis was performed using the Prism 9 software (GraphPad, San Diego, CA, USA). Data are presented as means (± SD and/or range), unless they are specified. Analyses of the gene expression data are based on delta-CT values, and the results are presented as relative (log/non-linear) fold changes using scatter plots. All other linear data are presented as scatter or bar graphs. Normality testing was performed via the Shapiro–Wilk test. The one-way analysis of variance (ANOVA), followed by a post hoc Tukey’s test, was applied, and statistical significance was set at *p* < 0.05.

## 3. Results

### 3.1. Functionalized MEM Supported Cell Growth and Function

**Figure 1:**
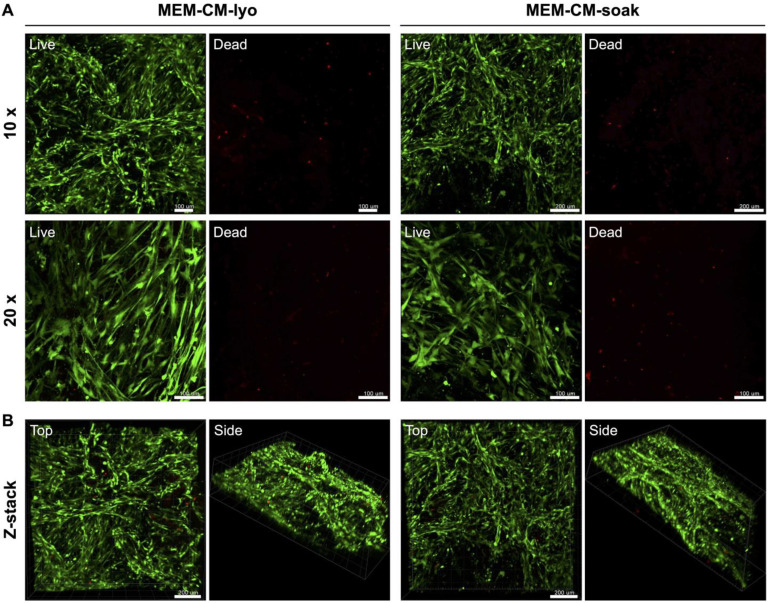
**Evaluation of cell viability on MEM-CM.** Representative 2D (**A**) and 3D (**B**) confocal images of rMSC viability (live/dead assay) on CM functionalized MEM either soaked (CM-SOAK) or lyophilized (CM-LYO) after 3 days. Scale bars: 100 μm (A) and 200 μm (B).

The live/dead assay revealed the high viability of rMSC on the MEM-CM, i.e., CM-LYO and CM-SOAK (Figure 1A), both of which were similar to that on the native MEM. The stacking of z-sections revealed the 3D migration of cells between the fibrillar network (pores) of the MEM (Figure 1B). The viability of rMSC on native MEM was also confirmed just prior to in vivo implantation (Supplementary Figure S2). With regard to cell function, the qPCR bioassay showed a significant upregulation of osteogenic gene markers in the BMSC exposed to CM vs. those in GM in monolayer cultures (Supplementary Figure S3). In the 3D cultures, i.e., BMSC seeded on native or functionalized MEM, a remarkable upregulation of all osteogenic genes was observed compared to cells in monolayer cultures, regardless of MEM functionalization. Thus, in 3D cultures, the effect of the MEM itself on the BMSC confounded the effects of MEM-CM. Although it was not statistically significant, a trend for enhanced gene expression was observed in the CM-LYO vs. CM-SOAK MEM-CM (Supplementary Figure S3).

### 3.2. CM-LYO Enhanced In Vivo New Bone Formation More than Other Treatments Did

**Figure 2:**
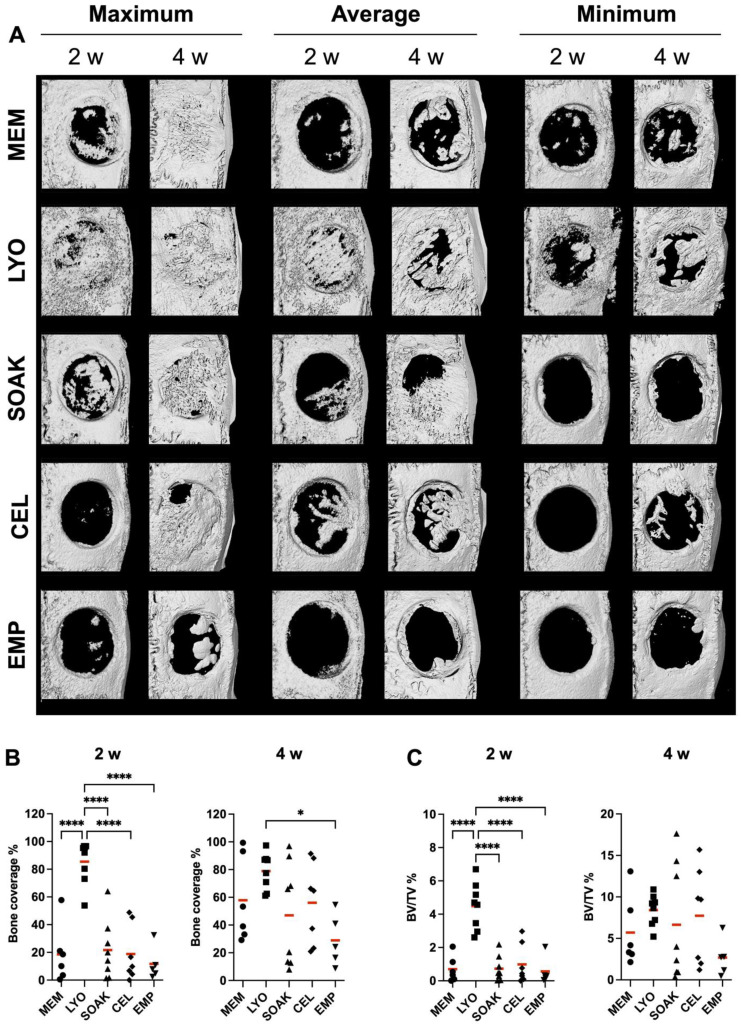
**μCT analysis.** (**A**) Representative reconstructed μCT images after 2 (in vivo) and 4 weeks (ex vivo) showing maximum, average and minimum bone formation in the different groups. Quantification of bone coverage (**B**) and bone volume per tissue volume (BV/TV) (**C**) in the different groups. MEM, native membrane; LYO, lyophilized membrane with conditioned media; SOAK, soaked membrane with conditioned media; CEL, membrane with rMSC; EMP, empty defects. Data represent means (n ≥ 5). * *p* < 0.05, **** *p* < 0.001.

**Figure 3:**
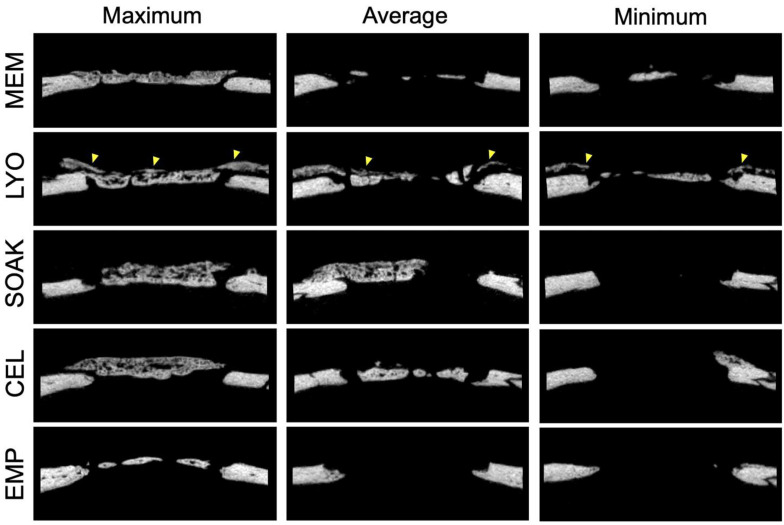
**μCT analysis (continued).** Representative μCT images of central slices at 4 weeks showing maximum, average and minimum bone formation in the different groups. Mineralization within the membrane component is indicated by yellow arrows in the LYO group representing hybrid bone and/or mineralized MEM fibers. MEM, native membrane; LYO, lyophilized membrane with conditioned media; SOAK, soaked membrane with conditioned media; CEL, membrane with rMSC; EMP, empty defects.

All animals included in the experiment recovered from the surgery, and no adverse events were observed. After 2 weeks, µCT revealed a significantly greater coverage of bone defects in the CM-LYO (85.5 ± 15.49%) vs. that in the CM-SOAK group (21.67 ± 21.13%; *p* < 0.001), suggesting a clear benefit of lyophilization (Figure 2A,B). Moreover, the bone coverage was also greater in CM-LYO vs. that in the CEL (18.81 ± 20.03%; *p* < 0.001), native MEM (18.57 ± 20.83%; *p* < 0.001) and empty groups (11.67 ± 12.02%; *p* < 0.001). A similar trend was observed with regard to BV/TV in CM-LYO (4.48 ± 1.4%) vs. CM-SOAK (0.72 ± 0.74; *p* < 0.001), CEL (0.98 ± 1.17; *p* < 0.001), native MEM (0.69 ± 0.77; *p* < 0.001) and empty groups (0.56 ± 0.83; *p* < 0.001) (Figure 2B). Early mineralization within the membrane compartment occurred more frequently in CM-LYO (8/8) vs. in the CM-SOAK (0/8), CEL (1/7) and MEM (2/6) groups (Figure 3).

After 4 weeks, only CM-LYO (78.9 ± 13.08%) showed significantly greater bone coverage than the untreated control group did (28.99 ± 18.64%; *p* = 0.027); those of the CM-SOAK (46.97 ± 37.12%), CEL (56.07 ± 29.22%) and native MEM groups (57.89 ± 30.9%) were similar. No differences in BV/TV were observed in CM-LYO (8.41 ± 1.87%) vs. that in the CM-SOAK (6.64 ± 7.01%; *p* = 0.947), CEL (7.72 ± 5.78%; *p* = 0.99), native MEM (5.7 ± 4.21%; *p* = 0.83) and empty groups (2.68 ± 2.24%; *p* = 0.25). Notably, the smallest intra-group variation was observed in the CM-LYO group (Figure 2A,B and Figure 3).

### 3.3. CM-LYO Promoted Histological New Bone Formation Better than Other Treatments Did

**Figure 4:**
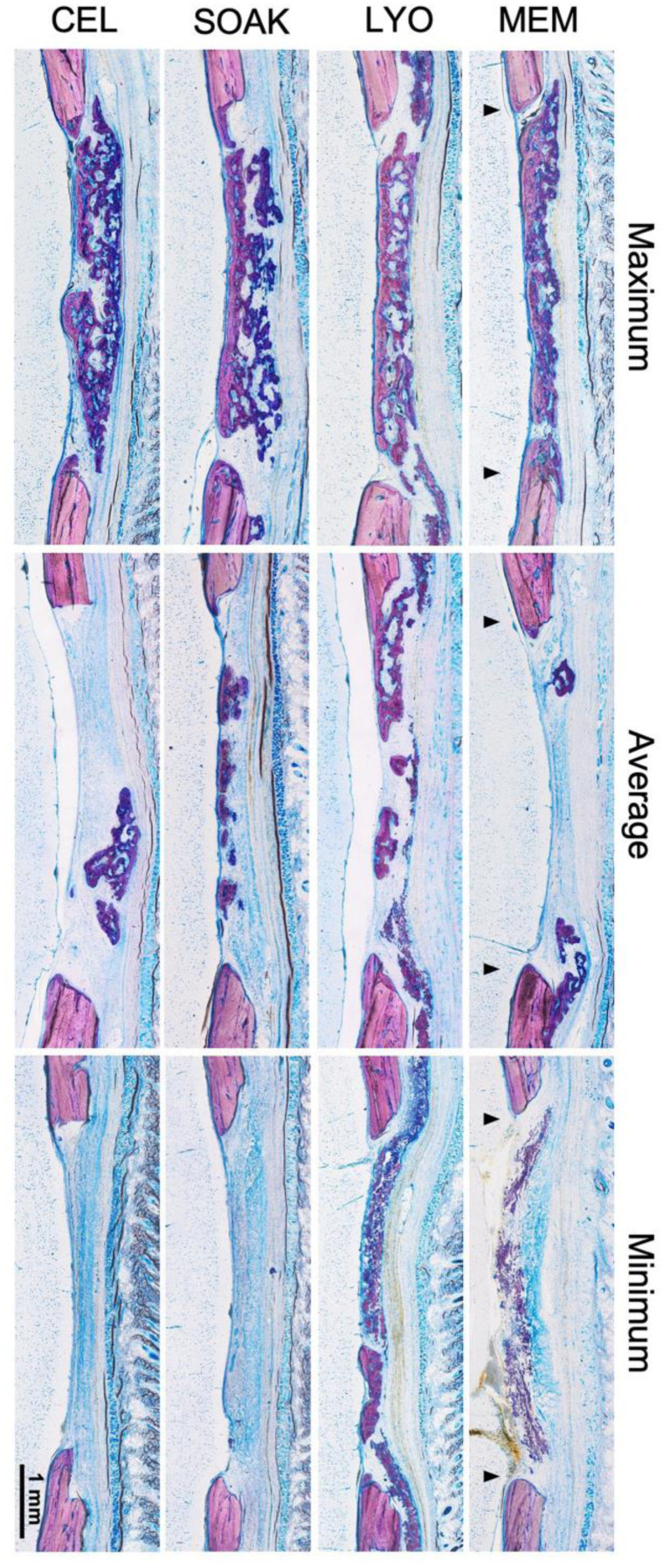
**Histological analysis.** Representative histological images after 4 weeks showing maximum, average and minimum bone regeneration in the different groups. Arrows indicate the defect edges. MEM, native membrane; LYO, lyophilized membrane with conditioned media; SOAK, soaked membrane with conditioned media; CEL, membrane with rMSC. Scale bar: 1 mm.

**Figure 5:**
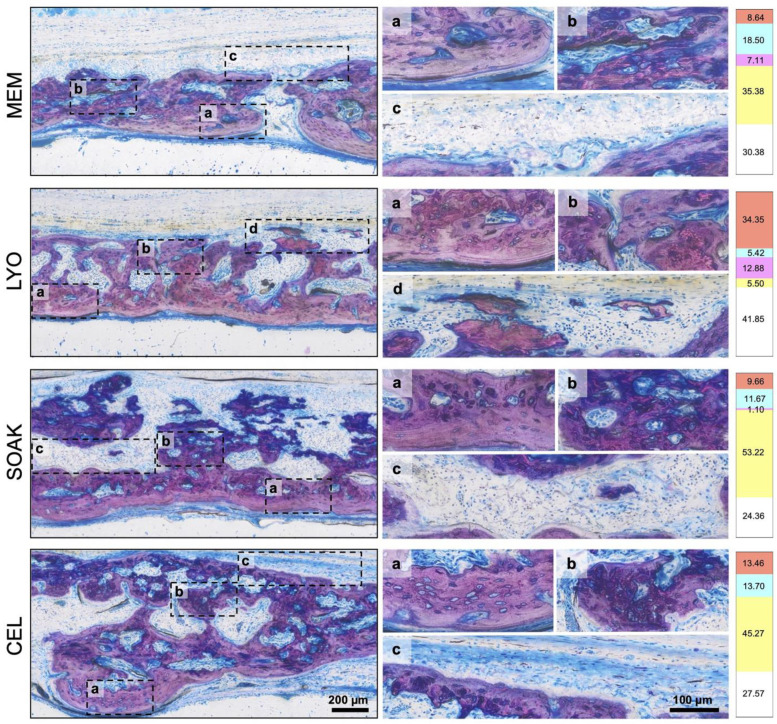
**Histological analysis (continued).** Representative histological images at higher magnification showing the different analyzed tissues in the experimental groups. The left panel shows a region of interest in each group with outlined sub-regions, which are enlarged in the right panel (scale bars: left panel 200 μm; right panel 100 μm). Each sub-region shows a specific tissue type indicated by letters (a–d). Numbers in the far right panel indicate relative percentages of tissue area in each group (for the whole group) based on color coding. Letters and colors indicate each tissue type: new bone (a, red), hybrid bone (b, cyan), residual membrane (c, yellow) and mineralized fibers (d, pink). White color in the far right panel indicates soft tissue areas.

**Figure 6:**
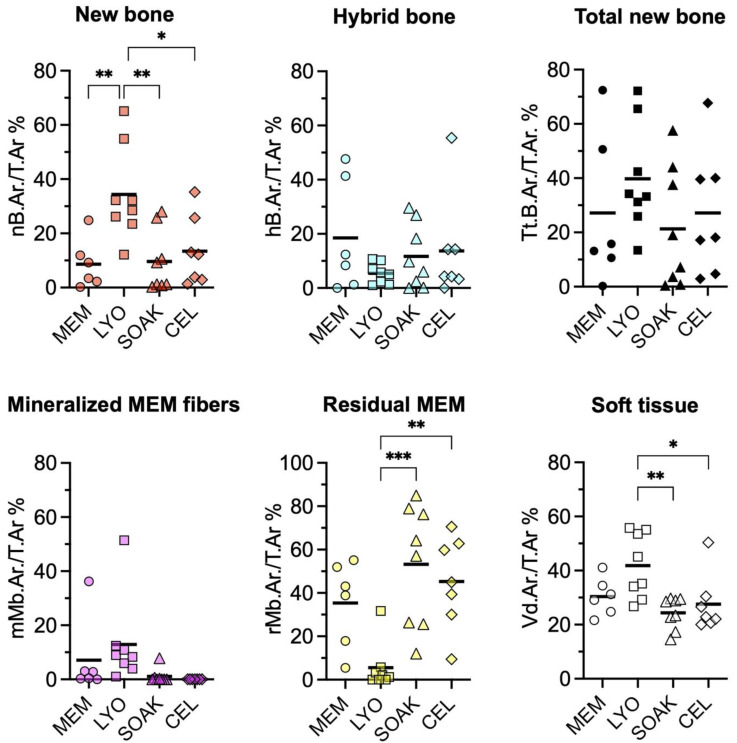
**Histomorphometry of central defect regions.** Quantification of histomorphometric parameters: T.Ar., Total Area; nB.Ar., New Bone Area; hB.Ar., Hybrid Bone Area; Tt.B.Ar., Total New Bone Area (New Bone + Hybrid Bone Area); mMb.Ar., Mineralized Membrane Area; rMb.Ar., Residual Membrane Area; Vd.Ar., Void Area; MEM, native membrane; LYO, lyophilized membrane with conditioned media; SOAK, soaked membrane with conditioned media; CEL, membrane with rMSC. Data represent means (n ≥ 5). * *p* < 0.05, ** *p* < 0.01, *** *p* < 0.001.

After 4 weeks, all the groups revealed a heterogeneous histological pattern combining the following tissue components: regular new bone (without incorporated MEM fibers), hybrid new bone (with incorporated MEM fibers), mineralized MEM fibers, residual MEM and soft tissues (Figure 4 and Figure 5). New bone was typically seen at the base of the defect towards the dura, i.e., outside the MEM compartment, characterized by well-structured woven bone (dark pink) and enclosed by layers of parallel-fibered bone (light pink) and osteoid matrix (grey). Adjacent to this newly formed bone, areas with a hybrid pattern characterized by the presence of immature woven bone and incorporated collagen fibers from the MEM (pink) were evident, indicating that some new bone formation occurred *within* the MEM compartment. In some cases, the MEM fibers appeared to be mineralized and formed bridges to the woven bone, while in other instances we observed mineralized ‘free-standing’ fibers (without surrounding woven bone) or remnant collagen fibers (unmineralized). Hybrid bone also appeared to vary based on the degree of mineralization of the embedded MEM fibers; the orientation of fibers followed the structure of the MEM. These different tissue types were observed in all the experimental groups, albeit in different proportions, as revealed in the histomorphometric analysis (Figure 5 and Figure 6).

The quantification of tissues in the central defect area revealed significantly greater amount of new bone in CM-LYO (34.35 ± 17.27%) vs. that in the CM-SOAK (9.66 ± 11.36%; *p* = 0.005), native MEM (8.63 ± 9.09%; *p* = 0.007) and CEL groups (13.46 ± 12.76%; *p* = 0.025) (Figure 6). Conversely, CM-LYO revealed the least amount of hybrid bone (5.42 ± 3.77%) vs. that in the CM-SOAK (11.67 ± 11.89%), MEM (18.5 ± 20.74%) and CEL groups (13.7 ± 19.23%), although this was not statistically significant (*p* = 0.43). The quantification of total new bone (new bone + hybrid bone) revealed a non-significant trend (*p* = 0.45) in CM-LYO (39.77 ± 19.85%) vs. that in the CM-SOAK (21.33 ± 22.25%), MEM (27.14 ± 28.02%) and CEL groups (27.16 ± 23.24%). CM-LYO also revealed the greatest area of mineralized MEM fibers (12.88 ± 16.01%) and the least area of residual MEM (5.5 ± 10.79%) vs. that of the other groups; the latter comparison was statistically significant (*p* < 0.001) (Figure 6). The intra-group variations, particularly for new bone, total new bone and residual MEM areas were relatively large. The quantification of tissue fractions in the defect edge areas revealed similar trends for the total new bone, mineralized MEM fibers and residual MEM between the groups (Supplementary Figure S4).

### 3.4. Structural, Mechanical and Compositional Differences Were Observed between Regular New Bone and Hybrid New Bone

**Figure 7:**
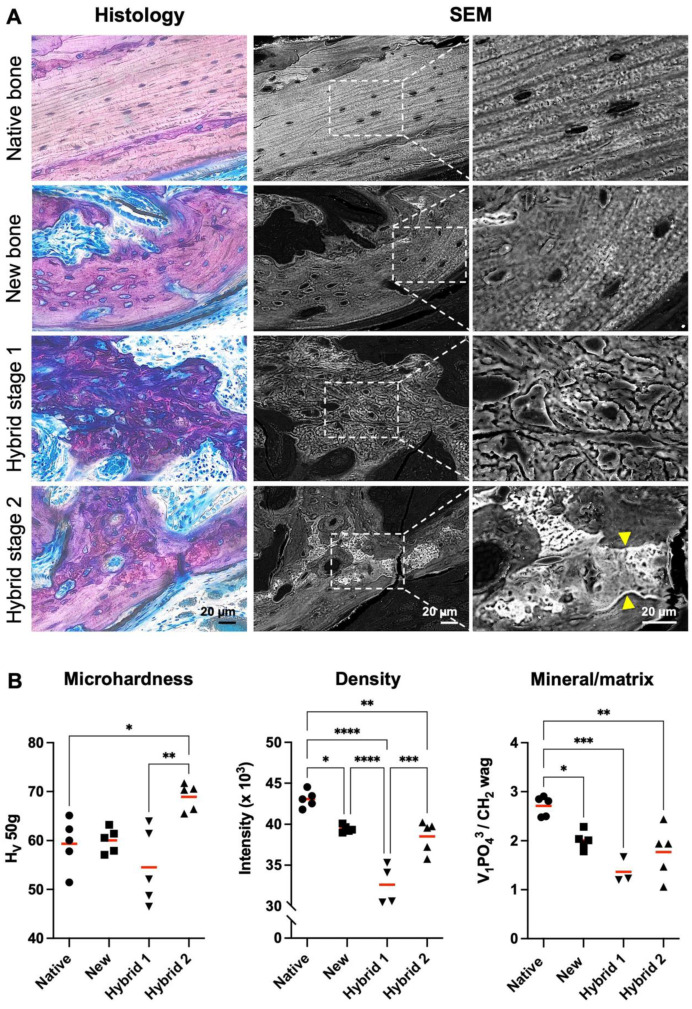
**Characterization of new and hybrid bone.** (**A**) Representative histological and corresponding scanning electron microscopy (SEM) images of native calvarial bone, new bone, hybrid stage 1 and hybrid stage 2 bones. Hybrid stage 1 and stage 2 bones are differentiated by degree of mineralization reflected by brightness in SEM images (a brighter area = more mineralized bone). Yellow arrows indicate cement lines characteristic of bone remodeling. Scale bars: 20 μm. (**B**) Quantification of microhardness (Vickers test), relative bone density (μCT), and mineral/matrix ratio (Raman spectroscopy) in native calvarial bone (Native), new bone (New), hybrid bone at stage 1 (Hybrid 1) and hybrid bone at stage 2 (Hybrid 2) in representative histological sections from each group (n ≥ 3 per group). Hv, Vickers hardness value; *v*_1_ PO_4_^3^, *v*_1_ phosphate peak; FWHM^−1^, full peak width at half maximum intensity for *v*_1_ PO_4_^3^ peak. Data represent means. * *p* < 0.05, ** *p* < 0.01, *** *p* = 0.001, **** *p* < 0.001.

The SEM analysis of the ultrastructure of new bone tissues confirmed that new bone (without incorporated MEM fibers) was most similar to the native calvaria bone, while hybrid bone (with incorporated MEM fibers) was more heterogenous. Detailed SEM analysis of hybrid bone revealed clear differences based on the degree of mineralization of the incorporated MEM fibers. Accordingly, the hybrid bone was further categorized as stage 1 (early stage, less mature and moderately mineralized) or stage 2 hybrid bone (later stage, more mature and highly mineralized) (Figure 7A). In the corresponding histology and SEM, stage 2 hybrid bone revealed evidence of resorption (cement lines) and remodeling, with the associated new bone deposition enveloping the hybrid bone (Figure 7A).

The quantitative analysis of the regular and hybrid new bone tissues was also performed based on their microhardness (Vickers test) and composition (µCT and Raman spectroscopy), in comparison to the native calvaria bone. Mechanical loading by Vickers indentation revealed significantly greater hardness in stage 2 vs. that in stage 1 hybrid bone and new bone (*p* = 0.002; Figure 7B). The bone density analysis demonstrated similar densities in the stage 2 hybrid bone and new bone, but a significantly lower density in stage 1 hybrid bone (*p* < 0.001; Figure 7B). No significant differences were observed in the mineral/matrix ratio (*v*_1_ PO_4_^3^/CH_2_ wag) (Figure 7B) or crystallinity (*v*_1_ PO_4_^3^ FWHM^−1^) between the new bone and stage 1 and 2 hybrid bones. A trend for lower mineral/matrix ratio was observed in stage 1 vs. stage 2 hybrid bone and new bone (*p* = 0.07). However, mineral/matrix ratios of all three tissues were significantly lower than that of native bone (Figure 7B). Representative Raman spectra are presented in Supplementary Figure S5**.**

### 3.5. Qualitative Proteomic Analysis of Lyophilized CM Revealed Enrichment of Biological Processes Related to Bone Formation

The proteomic analysis revealed 2684 proteins in the lyophilized pooled CM, of which 255 proteins were involved in selected biological processes related to bone formation (Table 1, Supplementary Table S2). Among the classical growth factors, transforming growth factor beta-1 (TGFβ1), TGFβ2, BMP1, platelet derived growth factor subunit-A (PDGFA), vascular endothelial growth factor-C (VEGFC), insulin-like growth factor-2 (IGF2), c-type lectin domain containing 11A or stem cell growth factor (CLEC11A/SCGF) and colony stimulating factor-1 (CSF1) were identified. Several proteins related to angiogenesis (VEGF-C, von Willebrand factor (VWF), vascular cell adhesion molecule-1 (VCAM1) and platelet endothelial cell adhesion molecule-1 (PECAM1), etc.) and ECM (collagens, laminins, fibronectin, etc.) were also identified in the CM.

**Figure 8:**
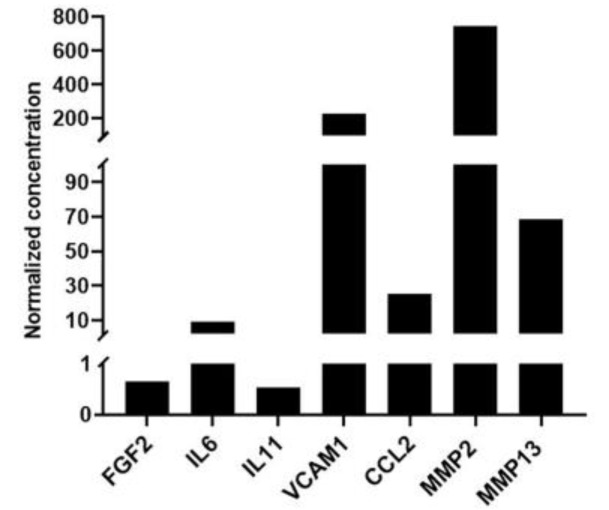
**Multiplex immunoassay.** Normalized concentrations of cytokines (pg cytokine/μg total protein) detected in CM using a human bone metabolism array (Supplementary Table S3). Data represent means of 4 technical replicates of a single sample of pooled CM (3 MSC donors).

The concentrations of selected bone-related cytokines in the lyophilized CM were further determined using a multiplex immunoassay; of the 31 array cytokines, 7 were present at detectable concentrations (Figure 8). Consistent with the proteomic analysis, matrix metalloproteinases-2 (MMP2) and -13 (MMP13), interleukins-6 (IL6) and -11 (IL11) and VCAM1 were detected in the pooled CM.

## 4. Discussion

Cell-free strategies using MSC-CM are emerging as cost-effective, ‘off-the-shelf’ alternatives to MSC transplantation for the regeneration of bone defects [17]. In the present study, we tested the efficacy of CM-functionalized MEM (CM-LYO, CM-SOAK) vs. that of native MEM or MEM seeded with allogeneic rMSC (CEL) for GBR in critical size rat calvaria defects. The main finding was that a trend for enhanced bone regeneration was observed in the CM-LYO group compared to the CM-SOAK, native MEM and CEL groups based on µCT and histological analysis.

The secretome/CM of MSC contains a plethora of different proteins, including growth factors, cytokines, chemokines and cell adhesion molecules, as well as lipids, nucleic acids and extracellular vesicles, which promote tissue healing and regeneration [45,46,47,48]. Consistent with previous reports [16], the data from the present study show that the exposure of BMSC to CM resulted in a significant upregulation of osteogenic gene markers. Additionally, we have recently reported that CM contain several antiapoptotic and antioxidative factors, which may inhibit apoptosis and/or promote cell survival [49]. From a clinical perspective, CM delivery presents clear advantages compared to implementing autologous cell therapies, since it is easier, cheaper and enables large-scale production [50,51]. Moreover, its application may be under less stringent regulation compared to that of cell therapies, which may facilitate faster clinical translation.

In the present study, MSC cultured in ‘xeno-free’ HPL-supplemented media were used for CM preparation. Most studies thus far have investigated CM from MSC cultured in ‘xenogeneic’, i.e., FBS-supplemented media, both in vitro [16] and in vivo (Supplementary Table S4). However, the exclusion of animal-derived supplements in MSC cultures is important for clinical translation and is in fact recommended by regulatory authorities [52]. Pooled HPL has been identified as the optimal xeno-free supplement for clinical grade MSC cultures [53], with particular benefits for osteogenic differentiation [27,54]. Indeed, the type of supplement can influence the composition and efficacy of the CM [55,56]. A few studies have investigated the composition of CM from HPL- vs. FBS-cultured MSC [47,57,58]. Recent evidence suggests that CM from HPL-cultured MSC are more ‘enriched’ in certain growth factors related to wound healing, angiogenesis and extra-cellular matrix (ECM) production [58], which may further promote their in vivo regenerative potential.

The in vivo applications of MSC-CM for bone regeneration have recently been reviewed [17] and are summarized in Supplementary Table S4. All the studies reported superior outcomes in bone defects treated with CM vs. those of control treatments in experimental in vivo investigations (mainly in rodent or rabbit models), although one study in a canine model also reported superior periodontal regeneration when comparing CM vs. PBS [59]. While a majority of the studies applied CM to bone defects using biomaterials, interestingly, CM also promoted regeneration when injected locally [60,61] and systemically [62] in challenging rodent models. Overall, while the current literature supports the use of CM for bone regeneration, certain aspects of this strategy remain unclear, such as, the optimal method/biomaterial for CM delivery and the optimal method of biomaterial functionalization for the best in vivo efficacy.

CM have been delivered using different biomaterials including collagen sponges, hydrogels, bone substitutes, barrier membranes and other scaffolds (Supplementary Table S4). Specifically, barrier membranes such as poly(lactic-co-glycolic acid) [63] and collagen [64,65] have been used, given their ability to absorb and release biomolecules at regeneration sites [19,21]. These biomolecules have included bone-derived proteins [23,24], BMP2 [66,67,68], fibroblast growth factor-2 [69] and dexamethasone [70], as recently summarized [4]. With regard to the collagen membranes used in the present study (MEM), we have previously demonstrated their ability to adsorb growth factor, specifically TGFβ, activity from human biological material [25]. Furthermore, Qiu et al. [19] recently reported the application of MEM soaked with CM from human periodontal- or gingiva-derived MSC in rat periodontal defects; significantly greater bone formation was observed in the defects treated with MEM-CM vs. those treated with native MEM [65]. Together, these data suggest that MEM are efficient carriers of bioactive factors, including CM, for GBR applications.

To identify the optimal method of functionalization herein, MEM were treated with concentrated CM (~30-fold) by either soaking only (CM-SOAK) or soaking followed by lyophilization (CM-LYO). The application of concentrated CM is reported to enhance tissue regeneration as compared that of unconcentrated CM [71]. Moreover, lyophilization or ‘freeze drying’ is reported to preserve the biological activity of proteins, e.g., growth factors, and other biological components for long-term storage [72]. Following MEM functionalization, both CM-LYO and CM-SOAK showed high cell viability, suggesting that they could be rapidly populated by resident cells following in vivo implantation. In the context of growth factors, the lyophilization of BMP2 on scaffolds, as compared to soaking, has been shown to enhance in vivo release and bone regeneration [73]. Indeed, CM-LYO showed superior bone regeneration compared to CM-SOAK in the present study. The lyophilization process may have resulted in superior concentration/immobilization and the subsequent in vivo release of proteins on/from the MEM, thus enhancing bone formation [74,75]. To exclude any effect of lyophilization on the MEM properties, we later studied the effects of lyophilized MEM (with serum-free DMEM) in the same calvaria defect model; a similar histological pattern was seen for the lyophilized MEM group as in the present study, suggesting no significant effect of the lyophilization process (unpublished data). Since lyophilization is a well-established and GMP-compliant process, this strategy could offer new possibilities for ‘off-the-shelf’ CM-based therapies for GBR.

Allogeneic MSC transplantation has been proposed as an easier, more cost-effective, and equally safe and efficacious alternative to autologous cell therapy [76]. This is based on the unique ability of MSC to modulate immune responses and avoid detection/rejection in dissimilar hosts [77]. Comparable or superior outcomes have been reported in in vivo models of bone regeneration when the researchers were using allogeneic vs. autologous cells [78,79,80,81,82]. In the present study, MEM seeded with pooled non-autologous rMSC (CEL) from syngeneic donor rats were also transplanted into calvarial defects. Trends of greater bone regeneration were observed in CM-LYO vs. that of the CEL-treated defects, which is consistent with previous reports of using CM and the corresponding cells, i.e., human BMSC [83], stem cells from human exfoliated deciduous teeth (SHED) [84] or rat adipose MSC [85]. Indeed, the present study used an inbred/syngeneic strain of rats, where the genetic diversity between the individuals, i.e., donor and recipient rats, was limited, and therefore, the cell source may not be strictly allogeneic. Moreover, immunological reactions to allogeneic/xenogeneic cells may not be accurately reflected in simple rodent models [86]. Thus, the true efficacy of CM vs. that of allogeneic MSC should be verified in large animal models of bone regeneration.

Inherent clinical limitations of resorbable MEM are their poor dimensional stability and sub-optimal mechanical strength and stiffness, which may result in their collapse into the bony defect unless they are supported by a biomaterial scaffold [20]. To enhance their long-term rigidity and stability, the concept of in vivo “self-mineralizing” membranes has been reported [87,88]. In the present study, in vivo MEM mineralization was observed, especially in the CM-LYO-treated MEM. This phenomenon of in vivo mineralization of collagen MEM was previously described by Feher et al. and is attributed to a potentially cell-independent mechanism [38]. It is reported that the hydrophobic nature of collagen MEM can facilitate calcium binding and mineralization by enhanced protein adsorption [89]. In the present study, the CM-LYO group exhibited the greatest histological area fraction of mineralized MEM fibers and new bone (without MEM fibers). The presence of stage 2 hybrid bone around the mineralized MEM fibers clearly indicates that MEM mineralization proceeded to new bone formation within the MEM compartment. Therefore, it is reasonable to hypothesize that MEM mineralization may have contributed to the overall bone regeneration, especially in the CM-LYO group.

An interesting finding in the present study was the heterogeneous pattern of new bone formation based on whether MEM collagen fibers were incorporated or not into the newly formed bone, i.e., new bone formation *within* and *outside* the membrane compartment. Indeed, the new bone outside the membrane compartment was histologically most similar to the native calvarial bone. In contrast, the hybrid bone (within the membrane compartment) was notably different from the native bone. A further analysis revealed distinct stages of hybrid bone formation based on the degree of mineralization of the MEM fibers, i.e., stage 1 (less mineralized) and stage 2 (more mineralized). This most likely reflects the stage of maturation, since an increasing degree of mineralization is a known age-related change in mineralized tissue [90]. Consequently, stage 2 hybrid bone demonstrated significantly greater hardness—an important indicator of bone strength mainly determined by the degree of mineralization [91]—than the new bone and even the native calvarial bone did. However, both the hybrid bone (stage 1 and 2) and new bone revealed significantly lower mineral/matrix ratios vs. that of the native calvaria bone. These data are similar to previous comparisons between new and native bone in rat calvaria [92,93] and to recent studies of bone regeneration following cell [94] and/or scaffold implantation [95]. With regard to its ‘fate’, the hybrid bone, particularly stage 2, showed signs of remodeling and replacement by new bone. This could potentially explain the relatively lower area of hybrid bone and the greater area of new bone (without MEM fibers) in the CM-LYO group. Taken together, the current data suggest that the use of MEM in rat calvaria results in a combination of regular new bone and hybrid new bone characterized by the incorporation of MEM in the newly formed bone. While further time course studies are needed to determine the exact sequence of events (with regard to MEM mineralization and hybrid bone formation), it appears that the hybrid bone is ultimately remodeled and replaced by regular new bone, and this process may be accelerated in CM-LYO-treated MEM.

The proteomic composition of lyophilized pooled CM was analyzed as the potential basis for its in vivo effects. Indeed, previous studies have comprehensively described the general proteomic profile of MSC [47,96], therefore, in the present study, we focused only on bone-related processes. Several key growth factors (TGFβ1, TGFβ2, PDGFA, VEGFC, etc.) involved in biological processes relevant to bone regeneration (Table 1) were identified in MSC-CM. Correspondingly, the expressions of several osteogenesis-related genes were enhanced in human BMSC upon exposure to CM for 48 h (Supplementary Figure S3), suggesting a pro-osteogenic effect. Moreover, several proteins related to Wnt/β-catenin signaling, a key signaling pathway during osteoblastogenesis [97], and angiogenesis were enriched in CM. In the context of MEM, Wnt-related [98] and angiogenesis-related proteins [4], in addition to TGFβ [25], have been shown to adsorb to collagen, revealing the potential mechanisms of MEM-CM activity. Correspondingly, areas of active in vivo bone formation (Supplementary Figure S6) and angiogenesis were observed in the present study. However, no remarkable differences in these events could be detected between the groups, which could be due to the relatively late time point of the histological analysis (4 weeks). Therefore, no reliable correlations between the in vitro and in vivo data could be drawn. The inclusion of earlier time points (1–2 weeks) and immunohistochemical methods in future studies to detect specific cells/processes may reveal such associations and their effects on in vivo bone regeneration. Nevertheless, the present study supports the current evidence for the efficacy of MSC-CM for in vivo bone regeneration [59,60,61,62,63,64,65,99,100,101,102,103,104,105,106,107,108].

Some limitations of the present study must be acknowledged. Firstly, the proteomic analysis was performed using only pooled CM (3 MSC donors) and not CM from independent donor-MSC, thus precluding the assessment of donor related variations. Secondly, human-derived MSC-CM were compared to rat-derived MSC (from syngeneic rats) in vivo, which may have cofounded the findings. However, the use of human-derived MSC would necessitate the use of immunocompromised animals, while the use of rat-derived MSC-CM would limit the clinical relevance of the therapy. The intra-group variations in the in vivo data were relatively large, reflecting biological differences in the healing response between the animals. Nevertheless, the measures of central tendency were reliable enough to allow the detection of statistically significant differences between the groups. Finally, in vitro assessments of protein adsorption and ‘release’ from the different functionalized membranes (CM-LYO and CM-SOAK) in future studies could shed light on potential differences in their mechanisms of action and their in vivo effects on bone regeneration.

## 5. Conclusions

Application of CM-LYO-functionalized MEM revealed a trend for enhanced GBR in rat calvaria defects compared to that of conventional GBR (MEM alone) and cell therapy (MEM with rMSC). The regenerated tissues presented a combination of regular new bone and hybrid new bone characterized by bone formation within the membrane compartment and incorporation of MEM in the newly formed bone. Further research is needed to determine the functional properties of these new bone tissues in terms of supporting implant osseointegration and prosthetic loading in more clinically relevant animal models. Moreover, future refinements of the study design and methodology may reveal correlations between the proteome of CM and in vivo processes. In summary, functionalizing MEM with MSC-CM represents a clinically relevant, ‘off-the-shelf’ strategy to promote GBR.

## Figures and Tables

**Table 1:** Summary of proteins in lyophilized pooled CM representing selected biological processes related to bone formation.

Bone-Related Process (GO Term) and Associated Proteins Identified in CM (Gene Name)
**GO:0030198 Extracellular matrix organization**
63 proteins: MMP2, COL1A1, EMILIN1, COL18A1, PXDN, TNXB, PRDX4, COL5A2, ADAMTSL4, MMP1, COL4A2, MMP3, MMP8, POSTN, COL4A1, COL11A1, COL8A2, ADAMTSL1, CYR61, COL1A2, NID1, MMP13, COL15A1, TNFRSF11B, B4GALT1, FBLN1, VTN, ABI3BP, ADAMTSL2, PTX3, COL3A1, ECM2, CCDC80, COL14A1, RECK, OLFML2B, MATN2, COL5A1, TGFBI, APP, MMP9, COL4A5, ADAMTS2, NDNF, MMP20, OLFML2A, COL8A1, ADAMTS12, COL5A3, MMP14, COL16A1, PDGFRA, COL24A1, ADAMTS7, COL4A3, ELN, ADAMTS4, MATN3, ADAMTS1, COL2A1, SMOC1, COL10A1, ADAMTS5
**GO:0030509 BMP signaling pathway**
15 proteins: PDCD4, TGFB1, TWSG1, EXT1, USP15, COMP, TGFB2, MEGF8, SMAD4, ENG, FST, WNT5A, NOTCH2, RGMB, TGFB3
**GO:0030513 Positive Regulation of BMP signaling pathway**
10 proteins: TWSG1, UBE2O, ILK, SMAD4, ENG, NUMA1, CDH5, SCUBE3, NOTCH2, SULF1
**GO:0045667/9 Regulation of osteoblast differentiation (positive regulation)**
19 proteins: PPP3CA, PTK2, CTNNB1, CLIC1, PRKACA, PDLIM7, FBN2, JAG1, ILK, FERMT2, LTF, IL6ST, YAP1, SCUBE3, FAM20C, IL6, SMOC1, TMEM119, CTHRC1
**GO:0060349 Bone morphogenesis**
8 proteins: GLG1, EXT1, COMP, MMP13, PAPPA2, LTF, LTBP3, SFRP4
**GO:0046849 Bone remodeling**
4 proteins: RAB7A, NOTCH2, LTBP3, GJA1
**GO:0046850/GO:0045780 Regulation of bone remodeling (positive and negative regulation)**
9 proteins: TF, SPP1, SRC, TFRC, SYK, ITGB3, LTBP3, GREM1, MDK
**GO:0030282 Bone mineralization**
12 proteins: CLEC3B, MINPP1, COMP, SBDS, COL1A2, MMP13, ENPP1, LOX, LTBP3, ALPL, PTN, ALOX15
**GO:0030500/1 Regulation of bone mineralization (positive regulation)**
13 proteins: OMD, TGFB1, COMP, AHSG, FBN2, ECM1, ISG15, ENPP1, FAM20C, PTN, TGFB3, TMEM119, ATP2B1
**GO:0001503 Ossification**
34 proteins: COL1A1, CLEC3B, COL5A2, SPP1, CDH11, TWSG1, EXT1, EGFR, MINPP1, COMP, BMP1, AHSG, CLEC11A, COL11A1, EXT2, PDLIM7, OSTF1, CSF1, ECM1, CBFB, IGF2, LTF, FSTL3, GPLD1, MMP9, ADAMTS12, MMP14, ADAMTS7, PTN, LRRC17, ALOX15, STC1, COL2A1, TMEM119
**GO:0030278/GO:0045778 Regulation of ossification (positive regulation)**
7 proteins: MAPK1, TGFB2, PTPN11, CSF1, MAPK14, WNT5A, PTN
**GO:0016055 Wnt signaling pathway**
20 proteins: DDB1, SLC9A3R1, CTNNB1, CUL3, TAX1BP3, CPE, TGFB1I1, PTK7, DAB2, PLCG2, RECK, TMEM198, STRN, WNT5A, NXN, CTNND1, ROR1, SFRP4, WNT5B, DDX3X
**GO:0030111 Regulation of Wnt signaling pathway**
4 proteins: PPP2CA, PPP2R1A, APP, SNX3
**GO:0007219 Notch signaling pathway**
16 proteins: FAT4, GOT1, TGFB1, ADAM17, CFD, JAG1, ADAM10, POFUT1, ANXA4, APP, WDR12, SORBS2, NOTCH2, EPN1, IFT74, TGFBR2
**GO:0008593 Regulation of Notch signaling pathway**
6 proteins: POSTN, ADAM10, CD46, IL6ST, POFUT1, LFNG
**GO:0001525 Angiogenesis**
86 proteins: MMP2, ITGA5, PDCL3, CSPG4, HRG, SHC1, PTK2, COL18A1, PXDN, FN1, YWHAZ, THY1, EPHB3, BSG, ANXA2, PDCD6, COL4A2, HMOX1, ANGPTL4, MFGE8, CLIC4, PDGFA, APOD, MYDGF, NCL, COL4A1, CALD1, COL8A2, ACTG1, TYMP, UNC5B, NRP1, COL15A1, MYH9, NRP2, ERAP1, JAG1, CXCL8, ECM1, MAPK14, VEGFC, ITGAV, VWF, MCAM, ANG, VAV2, CCL2, PDCD10, ANGPT1, ENG, PECAM1, FAP, ITGA2B, PIK3CA, SYK, GLUL, AIMP1, ANPEP, SERPINE1, VCAM1, POFUT1, ADAM15, TGFBI, HSPG2, FLNA, WASF2, EPGN, NDNF, PDGFRB, VEGFA, COL8A1, PLXND1, MMP14, SRPX2, CCBE1, EPHB2, FLT4, ESM1, PARVA, EGFL7, NPR3, GREM1, TNFRSF12A, AAMP, EFNB2, CAV1

GO, gene ontology. A complete list of gene names is provided in Supplementary Table S2.

## Data Availability

Additional data are included in the Supplementary File and can further be made available by the authors upon request.

## Supplementary Materials

The following supporting information can be downloaded at: https://www.mdpi.com/article/10.3390/cells12050767/s1, Figure S1: Histomorphometry segmentation; Figure S2: Cell viability on MEM; Figure S3: Gene expression assay; Figure S4: Histomorphometry of side regions; Figure S5: Raman spectroscopy; Figure S6: Histology; Table S1: Real time PCR primers; Table S2: List of proteins in CM representing selected bone-related processes; Table S3: List of cytokines included in the human bone metabolism multiplex array (*n* = 31); Table S4: In vivo studies of CM for bone regeneration.

## References

[1] Benic G.I., Hammerle C.H. (2014). Horizontal bone augmentation by means of guided bone regeneration. Periodontol. 2000.

[2] Thoma D.S., Bienz S.P., Figuero E., Jung R.E., Sanz-Martin I. (2019). Efficacy of lateral bone augmentation performed simultaneously with dental implant placement: A systematic review and meta-analysis. J. Clin. Periodontol..

[3] Urban I.A., Montero E., Monje A., Sanz-Sanchez I. (2019). Effectiveness of vertical ridge augmentation interventions: A systematic review and meta-analysis. J. Clin. Periodontol..

[4] Omar O., Elgali I., Dahlin C., Thomsen P. (2019). Barrier membranes: More than the barrier effect?. J. Clin. Periodontol..

[5] Chappuis V., Rahman L., Buser R., Janner S.F.M., Belser U.C., Buser D. (2018). Effectiveness of Contour Augmentation with Guided Bone Regeneration: 10-Year Results. J. Dent. Res..

[6] Gimbel M., Ashley R.K., Sisodia M., Gabbay J.S., Wasson K.L., Heller J., Wilson L., Kawamoto H., Bradley J. (2007). Repair of alveolar cleft defects: Reduced morbidity with bone marrow stem cells in a resorbable matrix. J. Craniofacial Surg..

[7] Shanbhag S., Suliman S., Pandis N., Stavropoulos A., Sanz M., Mustafa K. (2019). Cell therapy for orofacial bone regeneration: A systematic review and meta-analysis. J. Clin. Periodontol..

[8] Sandor G.K., Numminen J., Wolff J., Thesleff T., Miettinen A., Tuovinen V.J., Mannerström B., Patrikoski M., Seppänen R., Miettinen S. (2014). Adipose stem cells used to reconstruct 13 cases with cranio- maxillofacial hard- tissue defects. Stem Cells Transl. Med..

[9] Gjerde C., Mustafa K., Hellem S., Rojewski M., Gjengedal H., Yassin M.A., Feng X., Skaale S., Berge T., Rosen A. (2018). Cell therapy induced regeneration of severely atrophied mandibular bone in a clinical trial. Stem Cell Res. Ther..

[10] Haumer A., Bourgine P.E., Occhetta P., Born G., Tasso R., Martin I. (2018). Delivery of cellular factors to regulate bone healing. Adv. Drug Deliv. Rev..

[11] Caplan A.I., Dennis J.E. (2006). Mesenchymal stem cells as trophic mediators. J. Cell Biochem..

[12] Gnecchi M., Danieli P., Malpasso G., Ciuffreda M.C. (2016). Paracrine Mechanisms of Mesenchymal Stem Cells in Tissue Repair. Methods Mol. Biol..

[13] Pittenger M.F., Discher D.E., Péault B.M., Phinney D.G., Hare J.M., Caplan A.I. (2019). Mesenchymal stem cell perspective: Cell biology to clinical progress. NPJ Regen. Med..

[14] Weiss A.R.R., Dahlke M.H. (2019). Immunomodulation by Mesenchymal Stem Cells (MSCs): Mechanisms of Action of Living, Apoptotic, and Dead MSCs. Front. Immunol..

[15] Bari E., Perteghella S., Di Silvestre D., Sorlini M., Catenacci L., Sorrenti M., Marrubini G., Rossi R., Tripodo G., Mauri P. (2018). Pilot Production of Mesenchymal Stem/Stromal Freeze-Dried Secretome for Cell-Free Regenerative Nanomedicine: A Validated GMP-Compliant Process. Cells.

[16] Veronesi F., Borsari V., Sartori M., Orciani M., Mattioli-Belmonte M., Fini M. (2018). The use of cell conditioned medium for musculoskeletal tissue regeneration. J. Cell Physiol..

[17] Benavides-Castellanos M.P., Garzon-Orjuela N., Linero I. (2020). Effectiveness of mesenchymal stem cell-conditioned medium in bone regeneration in animal and human models: A systematic review and meta-analysis. Cell Regen..

[18] Dahlin C., Linde A., Gottlow J., Nyman S. (1988). Healing of bone defects by guided tissue regeneration. Plast Reconstr. Surg..

[19] Elgali I., Omar O., Dahlin C., Thomsen P. (2017). Guided bone regeneration: Materials and biological mechanisms revisited. Eur. J. Oral Sci..

[20] Caballe-Serrano J., Munar-Frau A., Ortiz-Puigpelat O., Soto-Penaloza D., Penarrocha M., Hernandez-Alfaro F. (2018). On the search of the ideal barrier membrane for guided bone regeneration. J. Clin. Exp. Dent..

[21] Turri A., Elgali I., Vazirisani F., Johansson A., Emanuelsson L., Dahlin C., Thomsen P., Omar O. (2016). Guided bone regeneration is promoted by the molecular events in the membrane compartment. Biomaterials.

[22] Caballe-Serrano J., Sawada K., Miron R.J., Bosshardt D.D., Buser D., Gruber R. (2017). Collagen barrier membranes adsorb growth factors liberated from autogenous bone chips. Clin. Oral Implant. Res..

[23] Kuchler U., Rybaczek T., Dobask T., Heimel P., Tangl S., Klehm J., Menzel M., Gruber R. (2018). Bone-conditioned medium modulates the osteoconductive properties of collagen membranes in a rat calvaria defect model. Clin. Oral Implant. Res..

[24] Strauss F.J., Kuchler U., Kobatake R., Heimel P., Tangl S., Gruber R. (2021). Acid bone lysates reduce bone regeneration in rat calvaria defects. J. Biomed Mater. Res. A.

[25] Di Summa F., Kargarpour Z., Nasirzade J., Stahli A., Mitulovic G., Panic-Jankovic T., Koller V., Kaltenbach C., Müller H., Panahipour L. (2020). TGFbeta activity released from platelet-rich fibrin adsorbs to titanium surface and collagen membranes. Sci. Rep..

[26] Donos N., Dereka X., Mardas N. (2015). Experimental models for guided bone regeneration in healthy and medically compromised conditions. Periodontol 2000.

[27] Shanbhag S., Mohamed-Ahmed S., Lunde T.H.F., Suliman S., Bolstad A.I., Hervig T., Mustafa K. (2020). Influence of platelet storage time on human platelet lysates and platelet lysate-expanded mesenchymal stromal cells for bone tissue engineering. Stem Cell Res. Ther..

[28] Rojewski M.T., Lotfi R., Gjerde C., Mustafa K., Veronesi E., Ahmed A.B., Wiesneth M., Körper S., Sensebé L., Layrolle P. (2019). Translation of a standardized manufacturing protocol for mesenchymal stromal cells: A systematic comparison of validation and manufacturing data. Cytotherapy.

[29] Al-Sharabi N., Mustafa M., Ueda M., Xue Y., Mustafa K., Fristad I. (2017). Conditioned medium from human bone marrow stromal cells attenuates initial inflammatory reactions in dental pulp tissue. Dent. Traumatol..

[30] Peng Y., Xuan M., Zou J., Liu H., Zhuo Z., Wan Y., Cheng B. (2015). Freeze-dried rat bone marrow mesenchymal stem cell paracrine factors: A simplified novel material for skin wound therapy. Tissue Eng. Part A.

[31] Mohamed-Ahmed S., Fristad I., Lie S.A., Suliman S., Mustafa K., Vindenes H., Idris S.B. (2018). Adipose-derived and bone marrow mesenchymal stem cells: A donor-matched comparison. Stem Cell Res. Ther..

[32] Shanbhag S., Suliman S., Mohamed-Ahmed S., Kampleitner C., Hassan M.N., Heimel P., Dobsak T., Tangl S., Bolstad A.I., Mustafa K. (2021). Bone regeneration in rat calvarial defects using dissociated or spheroid mesenchymal stromal cells in scaffold-hydrogel constructs. Stem Cell Res. Ther..

[33] Yassin M.A., Leknes K.N., Pedersen T.O., Xing Z., Sun Y., Lie S.A., Finne-Wistrand A., Mustafa K. (2015). Cell seeding density is a critical determinant for copolymer scaffolds-induced bone regeneration. J. Biomed Mater. Res. A.

[34] Toriumi D.M., Raslan W.F., Friedman M., Tardy M.E. (1990). Histotoxicity of cyanoacrylate tissue adhesives. A comparative study. Arch. Otolaryngol. Head Neck. Surg..

[35] Rezende M.L., Cunha Pde O., Damante C.A., Santana A.C., Greghi S.L., Zangrando M.S. (2015). Cyanoacrylate Adhesive as an Alternative Tool for Membrane Fixation in Guided Tissue Regeneration. J. Contemp Dent. Pract..

[36] An Y.Z., Strauss F.J., Park J.Y., Shen Y.Q., Thoma D.S., Lee J.S. Membrane fixation enhances guided bone regeneration in standardized calvarial defects: A pre-clinical study. J. Clin. Periodontol..

[37] Urbaniak G., Plous S. (2013). Research Randomizer, version 4.0.

[38] Feher B., Apaza Alccayhuaman K.A., Strauss F.J., Lee J.S., Tangl S., Kuchler U., Gruber R. (2021). Osteoconductive properties of upside-down bilayer collagen membranes in rat calvarial defects. Int. J. Implant Dent..

[39] Boivin G., Bala Y., Doublier A., Farlay D., Ste-Marie L.G., Meunier P.J., Delmas P. (2008). The role of mineralization and organic matrix in the microhardness of bone tissue from controls and osteoporotic patients. Bone.

[40] Unal M., Ahmed R., Mahadevan-Jansen A., Nyman J.S. (2021). Compositional assessment of bone by Raman spectroscopy. Analyst.

[41] Cesaratto A., Lombardi J., Leona M. (2016). Tracking photo-degradation of triarylmethane dyes with surface-enhanced Raman spectroscopy. J. Raman Spectrosc..

[42] Aasebo E., Brenner A.K., Hernandez-Valladares M., Birkeland E., Berven F.S., Selheim F., Bruserud Ø. (2021). Proteomic Comparison of Bone Marrow Derived Osteoblasts and Mesenchymal Stem Cells. Int. J. Mol. Sci..

[43] Bahlas S., Damiati L.A., Al-Hazmi A.S., Pushparaj P.N. (2020). Decoding the Role of Sphingosine-1-Phosphate in Asthma and Other Respiratory System Diseases Using Next Generation Knowledge Discovery Platforms Coupled With Luminex Multiple Analyte Profiling Technology. Front. Cell Dev. Biol..

[44] Liao Y., Wang J., Jaehnig E.J., Shi Z., Zhang B. (2019). WebGestalt 2019: Gene set analysis toolkit with revamped UIs and APIs. Nucleic Acids Res..

[45] Maffioli E., Nonnis S., Angioni R., Santagata F., Cali B., Zanotti L., Negri A., Viola A., Tedeschi G. (2017). Proteomic analysis of the secretome of human bone marrow-derived mesenchymal stem cells primed by pro-inflammatory cytokines. J. Proteomics..

[46] Baberg F., Geyh S., Waldera-Lupa D., Stefanski A., Zilkens C., Haas R., Schroeder T., Stühler K. (2019). Secretome analysis of human bone marrow derived mesenchymal stromal cells. Biochim. Biophys. Acta Proteins Proteom..

[47] Kehl D., Generali M., Mallone A., Heller M., Uldry A.C., Cheng P., Gantenbein B., Hoerstrup S.P., Weber B. (2019). Proteomic analysis of human mesenchymal stromal cell secretomes: A systematic comparison of the angiogenic potential. NPJ Regen. Med..

[48] Shin S., Lee J., Kwon Y., Park K.S., Jeong J.H., Choi S.J., Bang S.I., Chang J.W., Lee C. (2021). Comparative Proteomic Analysis of the Mesenchymal Stem Cells Secretome from Adipose, Bone Marrow, Placenta and Wharton’s Jelly. Int. J. Mol. Sci..

[49] Saleem R., Mohamed-Ahmed S., Elnour R., Berggreen E., Mustafa K., Al-Sharabi N. (2021). Conditioned Medium from Bone Marrow Mesenchymal Stem Cells Restored Oxidative Stress-Related Impaired Osteogenic Differentiation. Int. J. Mol. Sci..

[50] Sagaradze G., Grigorieva O., Nimiritsky P., Basalova N., Kalinina N., Akopyan Z., Efimenko A. (2019). Conditioned Medium from Human Mesenchymal Stromal Cells: Towards the Clinical Translation. Int. J. Mol. Sci..

[51] Marolt Presen D., Traweger A., Gimona M., Redl H. (2019). Mesenchymal Stromal Cell-Based Bone Regeneration Therapies: From Cell Transplantation and Tissue Engineering to Therapeutic Secretomes and Extracellular Vesicles. Front. Bioeng. Biotechnol..

[52] Bieback K., Fernandez-Munoz B., Pati S., Schafer R. (2019). Gaps in the knowledge of human platelet lysate as a cell culture supplement for cell therapy: A joint publication from the AABB and the. Int. Soc. Cell Gene Ther. Transfus..

[53] Fekete N., Rojewski M.T., Lotfi R., Schrezenmeier H. (2014). Essential components for ex vivo proliferation of mesenchymal stromal cells. Tissue Eng. Part C Methods.

[54] Shanbhag S., Suliman S., Bolstad A.I., Stavropoulos A., Mustafa K. (2020). Xeno-Free Spheroids of Human Gingiva-Derived Progenitor Cells for Bone Tissue Engineering. Front. Bioeng. Biotechnol..

[55] Madrigal M., Rao K.S., Riordan N.H. (2014). A review of therapeutic effects of mesenchymal stem cell secretions and induction of secretory modification by different culture methods. J. Transl. Med..

[56] Nikolits I., Nebel S., Egger D., Kress S., Kasper C. (2021). Towards Physiologic Culture Approaches to Improve Standard Cultivation of Mesenchymal Stem Cells. Cells.

[57] Palombella S., Guiotto M., Higgins G.C., Applegate L.L., Raffoul W., Cherubino M., Hart A., Riehle M.O., di Summa P.G. (2020). Human platelet lysate as a potential clinical-translatable supplement to support the neurotrophic properties of human adipose-derived stem cells. Stem Cell Res. Ther..

[58] Kim S.N., Lee C.J., Nam J., Choi B., Chung E., Song S.U. (2021). The Effects of Human Bone Marrow-Derived Mesenchymal Stem Cell Conditioned Media Produced with Fetal Bovine Serum or Human Platelet Lysate on Skin Rejuvenation Characteristics. Int. J. Stem Cells.

[59] Inukai T., Katagiri W., Yoshimi R., Osugi M., Kawai T., Hibi H., Ueda M. (2013). Novel application of stem cell-derived factors for periodontal regeneration. Biochem Biophys Res. Commun..

[60] Xu J., Wang B., Sun Y., Wu T., Liu Y., Zhang J., Lee W.Y., Pan X., Chai Y., Li G. (2016). Human fetal mesenchymal stem cell secretome enhances bone consolidation in distraction osteogenesis. Stem Cell Res. Ther..

[61] Fujio M., Xing Z., Sharabi N., Xue Y., Yamamoto A., Hibi H., Ueda M., Fristad I., Mustafa K. (2017). Conditioned media from hypoxic-cultured human dental pulp cells promotes bone healing during distraction osteogenesis. J. Tissue Eng. Regen. Med..

[62] Ogata K., Katagiri W., Osugi M., Kawai T., Sugimura Y., Hibi H., Nakamura S., Ueda M. (2015). Evaluation of the therapeutic effects of conditioned media from mesenchymal stem cells in a rat bisphosphonate-related osteonecrosis of the jaw-like model. Bone.

[63] Tsuchiya S., Ohmori M., Hara K., Fujio M., Ikeno M., Hibi H., Ueda M. (2015). An Experimental Study on Guided Bone Regeneration Using a Polylactide-co-glycolide Membrane-Immobilized Conditioned Medium. Int. J. Oral Maxillofac. Implant..

[64] Diomede F., D’Aurora M., Gugliandolo A., Merciaro I., Orsini T., Gatta V., Piattelli A., Trubiani O., Mazzon E. (2018). Biofunctionalized Scaffold in Bone Tissue Repair. Int. J. Mol. Sci..

[65] Qiu J., Wang X., Zhou H., Zhang C., Wang Y., Huang J. (2020). Enhancement of periodontal tissue regeneration by conditioned media from gingiva-derived or periodontal ligament-derived mesenchymal stem cells: A comparative study in rats. Stem Cell Res. Ther..

[66] Lai C.H., Zhou L., Wang Z.L., Lu H.B., Gao Y. (2013). Use of a collagen membrane loaded with recombinant human bone morphogenetic protein-2 with collagen-binding domain for vertical guided bone regeneration. J. Periodontol..

[67] Chang Y.Y., Lee J.S., Kim M.S., Choi S.H., Chai J.K., Jung U.W. (2015). Comparison of collagen membrane and bone substitute as a carrier for rhBMP-2 in lateral onlay graft. Clin. Oral Implants Res..

[68] Jo J.Y., Jeong S.I., Shin Y.M., Kang S.S., Kim S.E., Jeong C.M., Huh J.-B. (2015). Sequential delivery of BMP-2 and BMP-7 for bone regeneration using a heparinized collagen membrane. Int. J. Oral Maxillofac. Surg..

[69] Furuhata M., Takayama T., Yamamoto T., Ozawa Y., Senoo M., Ozaki M., Yamano S., Sato S. (2021). Real-time assessment of guided bone regeneration in critical size mandibular bone defects in rats using collagen membranes with adjunct fibroblast growth factor-2. J. Dent. Sci..

[70] Piao Z.G., Kim J.S., Son J.S., Lee S.Y., Fang X.H., Oh J.S., You J.-S., Kim S.-G. (2014). Osteogenic evaluation of collagen membrane containing drug-loaded polymeric microparticles in a rat calvarial defect model. Tissue Eng. Part A.

[71] Nagata M., Iwasaki K., Akazawa K., Komaki M., Yokoyama N., Izumi Y., Morita I. (2017). Conditioned Medium from Periodontal Ligament Stem Cells Enhances Periodontal Regeneration. Tissue Eng. Part A.

[72] Steil L., Thiele T., Hammer E., Bux J., Kalus M., Völker U., Greinacher A. (2008). Proteomic characterization of freeze-dried human plasma: Providing treatment of bleeding disorders without the need for a cold chain. Transfusion.

[73] Zhao J., Wang S., Bao J., Sun X., Zhang X., Zhang X., Ye D., Wei J., Liu C., Jiang X. (2013). Trehalose maintains bioactivity and promotes sustained release of BMP-2 from lyophilized CDHA scaffolds for enhanced osteogenesis in vitro and in vivo. PLoS ONE.

[74] Bari E., Di Silvestre D., Mastracci L., Grillo F., Grisoli P., Marrubini G., Nardini M., Mastrogiacomo M., Sorlini M., Rossi R. (2020). GMP-compliant sponge-like dressing containing MSC lyo-secretome: Proteomic network of healing in a murine wound model. Eur. J. Pharm. Biopharm..

[75] Zhang C., Wang T., Zhang L., Chen P., Tang S., Chen A., Li M., Peng G., Gao H., Weng H. (2021). Combination of lyophilized adipose-derived stem cell concentrated conditioned medium and polysaccharide hydrogel in the inhibition of hypertrophic scarring. Stem Cell Res. Ther..

[76] Kot M., Baj-Krzyworzeka M., Szatanek R., Musial-Wysocka A., Suda-Szczurek M., Majka M. (2019). The Importance of HLA Assessment in “Off-the-Shelf” Allogeneic Mesenchymal Stem Cells Based-Therapies. Int. J. Mol. Sci..

[77] Ankrum J.A., Ong J.F., Karp J.M. (2014). Mesenchymal stem cells: Immune evasive, not immune privileged. Nat. Biotechnol..

[78] Kang S.H., Chung Y.G., Oh I.H., Kim Y.S., Min K.O., Chung J.Y. (2014). Bone regeneration potential of allogeneic or autogeneic mesenchymal stem cells loaded onto cancellous bone granules in a rabbit radial defect model. Cell Tissue Res..

[79] Mahalingam V.D., Behbahani-Nejad N., Horine S.V., Olsen T.J., Smietana M.J., Wojtys E.M., Wellik D.M., Arruda E.M., Larkin L.M. (2015). Allogeneic versus autologous derived cell sources for use in engineered bone-ligament-bone grafts in sheep anterior cruciate ligament repair. Tissue Eng. Part A.

[80] Rapp A.E., Bindl R., Erbacher A., Kruchen A., Rojewski M., Schrezenmeier H., Müller I., Ignatius A. (2018). Autologous Mesenchymal Stroma Cells Are Superior to Allogeneic Ones in Bone Defect Regeneration. Int. J. Mol. Sci..

[81] Maiti S.K., Shivakumar M.U., Mohan D., Kumar N., Singh K.P. (2018). Mesenchymal Stem Cells of Different Origin-Seeded Bioceramic Construct in Regeneration of Bone Defect in Rabbit. Tissue Eng. Regen. Med..

[82] Lopez-Fernandez A., Barro V., Ortiz-Hernandez M., Manzanares M.C., Vivas D., Vives J., Vélez R., Ginebra M.P., Aguirre M. (2020). Effect of Allogeneic Cell-Based Tissue-Engineered Treatments in a Sheep Osteonecrosis Model. Tissue Eng. Part A.

[83] Osugi M., Katagiri W., Yoshimi R., Inukai T., Hibi H., Ueda M. (2012). Conditioned media from mesenchymal stem cells enhanced bone regeneration in rat calvarial bone defects. Tissue Eng. Part A.

[84] Hiraki T., Kunimatsu R., Nakajima K., Abe T., Yamada S., Rikitake K., Tanimoto K. (2020). Stem cell-derived conditioned media from human exfoliated deciduous teeth promote bone regeneration. Oral Dis..

[85] Sanchooli T., Norouzian M., Ardeshirylajimi A., Ghoreishi S., Abdollahifar M., Nazarian H., Piryaei A. (2017). Adipose Derived Stem Cells Conditioned Media in Combination with Bioceramic-Collagen Scaffolds Improved Calvarial Bone Healing in Hypothyroid Rats. Iran. Red Crescent Med. J..

[86] Mehler V.J., Burns C., Moore M.L. (2019). Concise Review: Exploring Immunomodulatory Features of Mesenchymal Stromal Cells in Humanized Mouse Models. Stem Cells.

[87] Wang J., Qu Y., Chen C., Sun J., Pan H., Shao C., Tang R., Gu X. (2019). Fabrication of collagen membranes with different intrafibrillar mineralization degree as a potential use for GBR. Mater. Sci. Eng. C Mater. Biol. Appl..

[88] Li J., Yan J.F., Wan Q.Q., Shen M.J., Ma Y.X., Gu J.T., Gao P., Tang X.-Y., Yu F., Chen J.-H. (2021). Matrix stiffening by self-mineralizable guided bone regeneration. Acta Biomater..

[89] Vo T.N., Ekenseair A.K., Spicer P.P., Watson B.M., Tzouanas S.N., Roh T.T., Mikos A.G. (2015). In vitro and in vivo evaluation of self-mineralization and biocompatibility of injectable, dual-gelling hydrogels for bone tissue engineering. J. Control. Release.

[90] Bailey A.J., Sims T.J., Ebbesen E.N., Mansell J.P., Thomsen J.S., Mosekilde L. (1999). Age-related changes in the biochemical properties of human cancellous bone collagen: Relationship to bone strength. Calcif Tissue Int..

[91] Ibrahim A., Magliulo N., Groben J., Padilla A., Akbik F., Abdel Hamid Z. (2020). Hardness, an Important Indicator of Bone Quality, and the Role of Collagen in Bone Hardness. J. Funct. Biomater..

[92] Ahmed R., Law A.W.L., Cheung T.W., Lau C. (2018). Raman spectroscopy of bone composition during healing of subcritical calvarial defects. Biomed. Opt. Express.

[93] Ahmed R., Wang W., Zia A.W., Lau C. (2018). Collagen formation observed from healing calvarial defects with principal component analysis of Raman scattering. Analyst.

[94] Du F., Wang Q., Ouyang L., Wu H., Yang Z., Fu X., Liu X., Yan L., Cao Y., Xiao R. (2021). Comparison of concentrated fresh mononuclear cells and cultured mesenchymal stem cells from bone marrow for bone regeneration. Stem Cells Transl. Med..

[95] Omar O., Engstrand T., Kihlstrom Burenstam Linder L., Aberg J., Shah F.A., Palmquist A., Birgersson U., Elgali I., Pujari-Palmer M., Engqvist H. (2020). In situ bone regeneration of large cranial defects using synthetic ceramic implants with a tailored composition and design. Proc. Natl. Acad. Sci. USA.

[96] Kupcova Skalnikova H. (2013). Proteomic techniques for characterisation of mesenchymal stem cell secretome. Biochimie.

[97] Okamoto M., Udagawa N., Uehara S., Maeda K., Yamashita T., Nakamichi Y., Kato H., Saito N., Minami Y., Takahashi N. (2014). Noncanonical Wnt5a enhances Wnt/beta-catenin signaling during osteoblastogenesis. Sci. Rep..

[98] Hiraiwa T., Nakai Y., Yamada T.G., Tanimoto R., Kimura H., Matsumoto Y., Miki N., Hiroi N., Funahashi A. (2018). Quantitative analysis of sensitivity to a Wnt3a gradient in determination of the pole-to-pole axis of mitotic cells by using a microfluidic device. FEBS Open Bio..

[99] Linero I., Chaparro O. (2014). Paracrine effect of mesenchymal stem cells derived from human adipose tissue in bone regeneration. PLoS ONE.

[100] Wang K.X., Xu L.L., Rui Y.F., Huang S., Lin S.E., Xiong J.H., Li Y.-H., Lee W.Y.-W., Li G. (2015). The effects of secretion factors from umbilical cord derived mesenchymal stem cells on osteogenic differentiation of mesenchymal stem cells. PLoS ONE.

[101] Katagiri W., Sakaguchi K., Kawai T., Wakayama Y., Osugi M., Hibi H. (2017). A defined mix of cytokines mimics conditioned medium from cultures of bone marrow-derived mesenchymal stem cells and elicits bone regeneration. Cell Prolif..

[102] Katagiri W., Osugi M., Kawai T., Ueda M. (2013). Novel cell-free regeneration of bone using stem cell-derived growth factors. Int. J. Oral Maxillofac. Implant..

[103] Katagiri W., Kawai T., Osugi M., Sugimura-Wakayama Y., Sakaguchi K., Kojima T., Kobayashi T. (2017). Angiogenesis in newly regenerated bone by secretomes of human mesenchymal stem cells. Maxillofac. Plast Reconstr. Surg..

[104] Kawai T., Katagiri W., Osugi M., Sugimura Y., Hibi H., Ueda M. (2015). Secretomes from bone marrow-derived mesenchymal stromal cells enhance periodontal tissue regeneration. Cytotherapy.

[105] Wang C.Y., Yang H.B., Hsu H.S., Chen L.L., Tsai C.C., Tsai K.S., Yew T.-L., Kao Y.-H., Hung S.-C. (2012). Mesenchymal stem cell-conditioned medium facilitates angiogenesis and fracture healing in diabetic rats. J. Tissue Eng. Regen. Med..

[106] Ogisu K., Fujio M., Tsuchiya S., Tsuboi M., Qi C., Toyama N., Kamio H., Hibi H. (2020). Conditioned media from mesenchymal stromal cells and periodontal ligament fibroblasts under cyclic stretch stimulation promote bone healing in mouse calvarial defects. Cytotherapy.

[107] Diomede F., Gugliandolo A., Scionti D., Merciaro I., Cavalcanti M.F., Mazzon E. (2018). Biotherapeutic effect of gingival stem cells conditioned medium in bone tissue restoration. Int. J. Mol. Sci..

[108] Pranskunas M., Simoliunas E., Alksne M., Martin V., Gomes P.S., Puisys A., Kaupinis A., Juodzbalys G. (2021). Assessment of the Bone Healing Process Mediated by Periosteum-Derived Mesenchymal Stem Cells’ Secretome and a Xenogenic Bioceramic-An In Vivo Study in the Rabbit Critical Size Calvarial Defect Model. Materials.

