# Supplementary material for: Functionalizing Collagen Membranes with MSC-Conditioned Media Promotes Guided Bone Regeneration in Rat Calvarial Defects
Source: Cells. 2023 Feb 28;12(5):767. doi: 10.3390/cells12050767 (PMC10001262; doi:10.3390/cells12050767)
Supplement: Supplementary file 1 [file cells-12-00767-s001.zip › cells-2239213-SI.pdf]

Functionalizing collagen membranes with MSC conditioned media promotes guided bone regeneration in rat calvarial defects

Supplementary methods

Bone density analysis

To perform the measurements, in each region, the maximum intensity was measured. To estimate an average density in an area, the following filtering procedure was used. At the surface of an object in a µCT scan, an intensity gradient is present which would influence the average intensity in the region. Pores in the bone would further reduce the average intensity. Areas with an intensity lower than bone or a pixel wise intensity difference to the local median (radius 10 pixels, 134 µm) >2000 were set to their local maximum (radius 4 pixels, 53.6 µm). A greyscale open operation (radius 4 pixels, 53.6 µm) was then performed to flatten out the intensity of the remaining surface gradient and pores below the detection threshold. Pores based on the difference to median and areas below the bone threshold in the original MIP and were then set to 0 in the filtered image. Average intensity in a region was measured by calculating the average of all pixels with an intensity >0.

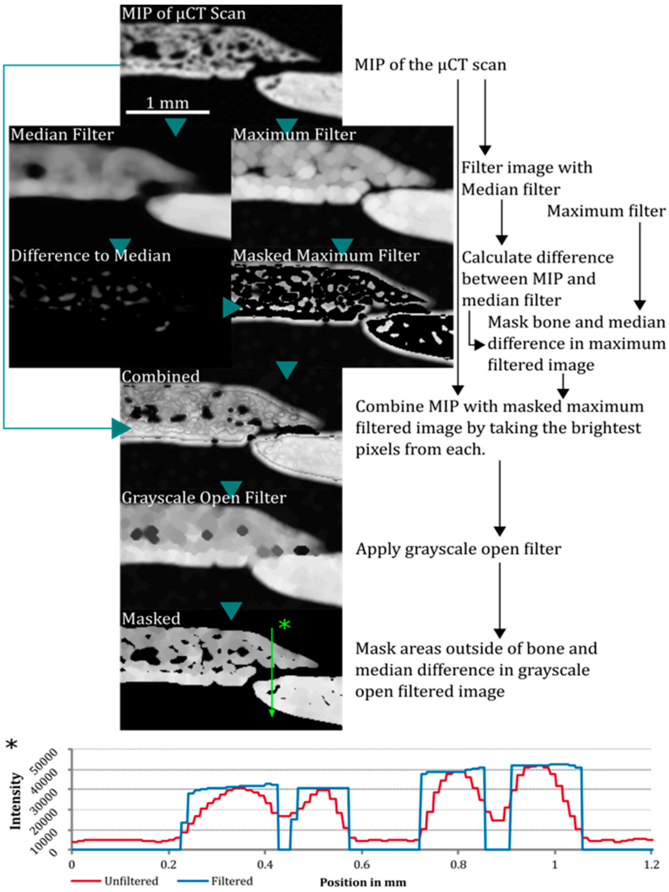

Step by step illustration of the filtering procedure. A maximum filter is applied to the MIP of the µCT scan. The filtered image is masked with the thresholded bone of the MIP and the difference between the MIP and a median filter. The masked image is combined with the MIP of the µCT, taking the highest value from the two images for each pixel. A grayscale open filter is applied to the combined image which is then masked outside of the bone and areas of high difference to the median. An intensity profile is plotted along the green arrow (*) comparing the unfiltered MIP with the final result of the filtration. This procedure removes the intensity gradient on the surface of structures in the µCT scan so an average intensity can be calculated in an area while intending to minimize the effect of the local geometry.

Liquid chromatography with tandem mass spectrometry (LC-MS/MS)

LC run (195 min)

Peptides were separated during a biphasic ACN gradient from two nanoflow UPLC pumps (flow rate of 200 nl/min) on a 50 cm analytical column (PepMap RSLC, 50cm x 75 µm ID EASY-spray column, packed with 2µm C18 beads). Solvent A and B were 0.1% TFA (vol/vol) in water and 100% ACN respectively. The gradient composition was 5%B during trapping (5min) followed by 5-8%B over 1 min, 8–25%B for the next 124min, 25-35%B over 30 min, and 35–85%B over 5min. Elution of very hydrophobic peptides and conditioning of the column were performed during 10 minutes isocratic elution with 85%B and 15 minutes isocratic conditioning with 5%B. Instrument control was through Thermo Scientific SII for Xcalibur 1.6.

High field asymmetric waveform ion mobility spectrometry (FAIMS)

The FAIMS Pro interface performs gas-phase fractionation, enabling preferred accumulation of multiply charged ions to maximize the efficiency of data-dependent acquisition (DDA) routines and increase proteome coverage. Short-ion residence time in the FAIMS Pro interface electrode assembly enables use of multiple CV settings in a single run to increase proteome coverage.

DDA with FAIMS

Peptides eluted from the column were detected in the Exploris 480 Mass Spectrometer with FAIMS enabled using three compensation voltages (CVs), -45V, -65V and -80V respectively, and "Advanced Peak Determination" on. During each CV, the mass spectrometer was operated in the DDA-mode (data-dependent-acquisition) to automatically switch between one full scan MS and MS/MS acquisition. Instrument control was through Orbitrap Exploris 480 Tune 3.1 and Xcalibur 4.4. The cycle time was maintained at 1.2s/CV (-45 and -65) or 0.8s/CV (-80). MS spectra were acquired in the scan range 375-1500 m/z with resolution R = 120 000 at m/z 200, automatic gain control (AGC) target of 3e6 and a maximum injection time (IT) at auto (depending on transient length in the orbitrap). The most intense eluting peptides with charge states 2 to 6 were sequentially isolated to standard target value (AGC, usually 1e5) or a maximum IT of 75 ms in the C-trap, and isolation width maintained at 1.6 m/z (quadrupole isolation), before fragmentation in the HCD (Higher-Energy Collision Dissociation. Fragmentation was performed with a normalized collision energy (NCE) of 30

%, and fragments were detected in the Orbitrap at a resolution of 15 000 at m/z 200, with first mass fixed at m/z 120. One MS/MS spectrum of a precursor mass was allowed before dynamic exclusion for 30s with “exclude isotopes” on. Lock-mass internal calibration was not enabled.

Ionsource parameter

The spray and ion-source parameters were as follows. Ion spray voltage = 2000V, no sheath and auxiliary gas flow, and capillary temperature = 275 °C.

Supplementary tables

Supplementary Table S1: Real time PCR primers

Gene (human)	TaqMan® Assay ID	Amplicon length
**Reference**		
GAPDH	Hs 02758991_g1	93
**Osteogenesis**		
RUNX2	Hs01047973_m1	86
BMP2	Hs00154192_m1	60
OPN (SPP1)	Hs00959010_m1	84
OCN (BGLAP)	Hs01587814_g1	138

GAPDH glyceraldehyde 3-phosphate dehydrogenase, RUNX2 runt-related transcription factor 2, BMP2 bone morphogenetic protein 2, COL1A2 Collagen type 1 alpha 2, OPN/SPP1 osteopontin, OCN/BGLAP osteocalcin.

Supplementary Table S2: List of proteins in CM representing selected bone-related processes (Table 1 in manuscript)

Gene symbol	Gene name
AAMP	angio associated migratory cell protein
ABI3BP	ABI family member 3 binding protein
ACD	ACD shelterin complex subunit and telomerase recruitment factor
ACTG1	actin gamma 1
ADAM10	ADAM metallopeptidase domain 10
ADAM15	ADAM metallopeptidase domain 15
ADAM17	ADAM metallopeptidase domain 17
ADAMTS1	ADAM metallopeptidase with thrombospondin type 1 motif 1
ADAMTS12	ADAM metallopeptidase with thrombospondin type 1 motif 12
ADAMTS13	ADAM metallopeptidase with thrombospondin type 1 motif 13
ADAMTS2	ADAM metallopeptidase with thrombospondin type 1 motif 2
ADAMTS4	ADAM metallopeptidase with thrombospondin type 1 motif 4
ADAMTS5	ADAM metallopeptidase with thrombospondin type 1 motif 5
ADAMTS7	ADAM metallopeptidase with thrombospondin type 1 motif 7
ADAMTSL1	ADAMTS like 1
ADAMTSL2	ADAMTS like 2
ADAMTSL4	ADAMTS like 4
AHSG	alpha 2-HS glycoprotein
AIMP1	aminoacyl tRNA synthetase complex interacting multifunctional protein 1
AKT1	AKT serine/threonine kinase 1
ALOX15	arachidonate 15-lipoxygenase
ALPL	alkaline phosphatase, biomineralization associated
ANG	angiogenin
ANGPT1	angiopoietin 1
ANGPTL4	angiopoietin like 4
ANGPTL6	angiopoietin like 6
ANPEP	alanyl aminopeptidase, membrane
ANXA2	annexin A2
ANXA4	annexin A4
ANXA6	annexin A6
APOD	apolipoprotein D
APOE	apolipoprotein E
APP	amyloid beta precursor protein
ARRB1	arrestin beta 1
ATP2B1	ATPase plasma membrane Ca2+ transporting 1
ATP6AP2	ATPase H+ transporting accessory protein 2
B4GALT1	beta-1,4-galactosyltransferase 1
BMP1	bone morphogenetic protein 1
BSG	basigin (Ok blood group)
BTF3P11	basic transcription factor 3 pseudogene 11

C1D	C1D nuclear receptor corepressor
CALD1	caldesmon 1
CAV1	caveolin 1
CBFB	core-binding factor subunit beta
CBS	cystathionine beta-synthase
CCBE1	collagen and calcium binding EGF domains 1
CCDC80	coiled-coil domain containing 80
CCL2	C-C motif chemokine ligand 2
CCN1	cellular communication network factor 1
CD46	CD46 molecule
CDC42	cell division cycle 42
CDH11	cadherin 11
CDH2	cadherin 2
CDH5	cadherin 5
CFD	complement factor D
CHD8	chromodomain helicase DNA binding protein 8
CLEC11A	C-type lectin domain containing 11A
CLEC3B	C-type lectin domain family 3 member B
CLIC1	chloride intracellular channel 1
CLIC4	chloride intracellular channel 4
COL10A1	collagen type X alpha 1 chain
COL11A1	collagen type XI alpha 1 chain
COL14A1	collagen type XIV alpha 1 chain
COL15A1	collagen type XV alpha 1 chain
COL16A1	collagen type XVI alpha 1 chain
COL18A1	collagen type XVIII alpha 1 chain
COL1A1	collagen type I alpha 1 chain
COL1A2	collagen type I alpha 2 chain
COL24A1	collagen type XXIV alpha 1 chain
COL2A1	collagen type II alpha 1 chain
COL3A1	collagen type III alpha 1 chain
COL4A1	collagen type IV alpha 1 chain
COL4A2	collagen type IV alpha 2 chain
COL4A3	collagen type IV alpha 3 chain
COL4A5	collagen type IV alpha 5 chain
COL5A1	collagen type V alpha 1 chain
COL5A2	collagen type V alpha 2 chain
COL5A3	collagen type V alpha 3 chain
COL8A1	collagen type VIII alpha 1 chain
COL8A2	collagen type VIII alpha 2 chain
COMP	cartilage oligomeric matrix protein
CPE	carboxypeptidase E
CSF1	colony stimulating factor 1

CSK	C-terminal Src kinase
CSPG4	chondroitin sulfate proteoglycan 4
CTHRC1	collagen triple helix repeat containing 1
CTNNB1	catenin beta 1
CTNND1	catenin delta 1
CUL3	cullin 3
CXCL8	C-X-C motif chemokine ligand 8
CXXC1	CXXC finger protein 1
DAB2	DAB adaptor protein 2
DDB1	damage specific DNA binding protein 1
DDX3X	DEAD-box helicase 3 X-linked
DKK3	dickkopf WNT signaling pathway inhibitor 3
DNAJB6	DnaJ heat shock protein family (Hsp40) member B6
DOCK5	dedicator of cytokinesis 5
DRAXIN	dorsal inhibitory axon guidance protein
ECM1	extracellular matrix protein 1
ECM2	extracellular matrix protein 2
EFNB2	ephrin B2
EGF	epidermal growth factor
EGFL7	EGF like domain multiple 7
EGFR	epidermal growth factor receptor
ELN	elastin
EMD	emerin
EMILIN1	elastin microfibril interfacer 1
ENG	endoglin
ENPP1	ectonucleotide pyrophosphatase/phosphodiesterase 1
EPGN	epithelial mitogen
EPHB2	EPH receptor B2
EPHB3	EPH receptor B3
EPN1	epsin 1
ERAP1	endoplasmic reticulum aminopeptidase 1
ESM1	endothelial cell specific molecule 1
EXT1	exostosin glycosyltransferase 1
EXT2	exostosin glycosyltransferase 2
F3	coagulation factor III, tissue factor
FAM20C	FAM20C golgi associated secretory pathway kinase
FAP	fibroblast activation protein alpha
FAT4	FAT atypical cadherin 4
FBLN1	fibulin 1
FBLN2	fibulin 2
FBN2	fibrillin 2
FERMT2	FERM domain containing kindlin 2
FLNA	filamin A

FLT4	fms related receptor tyrosine kinase 4
FN1	fibronectin 1
FST	follistatin
FSTL3	follistatin like 3
G3BP1	G3BP stress granule assembly factor 1
GJA1	gap junction protein alpha 1
GLG1	golgi glycoprotein 1
GLUL	glutamate-ammonia ligase
GMDS	GDP-mannose 4,6-dehydratase
GOLT1A	golgi transport 1A
GOLT1B	golgi transport 1B
GOT1	glutamic-oxaloacetic transaminase 1
GPLD1	glycosylphosphatidylinositol specific phospholipase D1
GREM1	gremlin 1, DAN family BMP antagonist
HMOX1	heme oxygenase 1
HRG	histidine rich glycoprotein
HSPG2	heparan sulfate proteoglycan 2
IFT74	intraflagellar transport 74
IGF2	insulin like growth factor 2
IGFBP2	insulin like growth factor binding protein 2
IGFBP4	insulin like growth factor binding protein 4
IGFBP6	insulin like growth factor binding protein 6
IL6	interleukin 6
IL6ST	interleukin 6 cytokine family signal transducer
ILK	integrin linked kinase
INPP5D	inositol polyphosphate-5-phosphatase D
INPPL1	inositol polyphosphate phosphatase like 1
ISG15	ISG15 ubiquitin like modifier
ITGA2B	integrin subunit alpha 2b
ITGA5	integrin subunit alpha 5
ITGAV	integrin subunit alpha V
ITGB3	integrin subunit beta 3
JAG1	jagged canonical Notch ligand 1
KMT2A	lysine methyltransferase 2A
KPNA1	karyopherin subunit alpha 1
LFNG	LFNG O-fucosylpeptide 3-beta-N-acetylglucosaminyltransferase
LOX	lysyl oxidase
LRP1	LDL receptor related protein 1
LRRC17	leucine rich repeat containing 17
LTBP2	latent transforming growth factor beta binding protein 2
LTBP3	latent transforming growth factor beta binding protein 3
LTF	lactotransferrin
MACF1	microtubule actin crosslinking factor 1

MAPK1	mitogen-activated protein kinase 1
MAPK14	mitogen-activated protein kinase 14
MATN2	matrilin 2
MATN3	matrilin 3
MCAM	melanoma cell adhesion molecule
MDK	midkine
MEGF8	multiple EGF like domains 8
MFGE8	milk fat globule EGF and factor V/VIII domain containing
MINPP1	multiple inositol-polyphosphate phosphatase 1
MMP1	matrix metallopeptidase 1
MMP13	matrix metallopeptidase 13
MMP14	matrix metallopeptidase 14
MMP2	matrix metallopeptidase 2
MMP20	matrix metallopeptidase 20
MMP25	matrix metallopeptidase 25
MMP3	matrix metallopeptidase 3
MMP8	matrix metallopeptidase 8
MMP9	matrix metallopeptidase 9
MRPS12	mitochondrial ribosomal protein S12
MYDGF	myeloid derived growth factor
MYH9	myosin heavy chain 9
NCL	nucleolin
NDNF	neuron derived neurotrophic factor
NELL2	neural EGFL like 2
NEO1	neogenin 1
NHERF1	NHERF family PDZ scaffold protein 1
NID1	nidogen 1
NOTCH2	notch receptor 2
NRG1	neuregulin 1
NRP1	neuropilin 1
NRP2	neuropilin 2
NUMA1	nuclear mitotic apparatus protein 1
NXN	nucleoredoxin
OLFML2A	olfactomedin like 2A
OLFML2B	olfactomedin like 2B
OMD	osteomodulin
OSTF1	osteoclast stimulating factor 1
PAPPA2	pappalysin 2
PARVA	parvin alpha
PDCD10	programmed cell death 10
PDCD4	programmed cell death 4
PDCD6	programmed cell death 6
PDCL3	phosducin like 3

PDE3B	phosphodiesterase 3B
PDGFA	platelet derived growth factor subunit A
PDGFRA	platelet derived growth factor receptor alpha
PDGFRB	platelet derived growth factor receptor beta
PDLIM7	PDZ and LIM domain 7
PECAM1	platelet and endothelial cell adhesion molecule 1
PFDN5	prefoldin subunit 5
PIK3CA	phosphatidylinositol-4,5-bisphosphate 3-kinase catalytic subunit alpha
PIN1	peptidylprolyl cis/trans isomerase, NIMA-interacting 1
PLCG2	phospholipase C gamma 2
PLXND1	plexin D1
POFUT1	protein O-fucosyltransferase 1
POSTN	periostin
PPM1A	protein phosphatase, Mg2+/Mn2+ dependent 1A
PPM1B	protein phosphatase, Mg2+/Mn2+ dependent 1B
PPP1CA	protein phosphatase 1 catalytic subunit alpha
PPP2CA	protein phosphatase 2 catalytic subunit alpha
PPP2R1A	protein phosphatase 2 scaffold subunit Aalpha
PPP3CA	protein phosphatase 3 catalytic subunit alpha
PRDX4	peroxiredoxin 4
PRKACA	protein kinase cAMP-activated catalytic subunit alpha
PTK2	protein tyrosine kinase 2
PTK7	protein tyrosine kinase 7 (inactive)
PTN	pleiotrophin
PTPN11	protein tyrosine phosphatase non-receptor type 11
PTPRU	protein tyrosine phosphatase receptor type U
PTX3	pentraxin 3
PXDN	peroxidasin
RAB7A	RAB7A, member RAS oncogene family
RAC1	Rac family small GTPase 1
RECK	reversion inducing cysteine rich protein with kazal motifs
RGMB	repulsive guidance molecule BMP co-receptor b
RHOA	ras homolog family member A
RNASE1	ribonuclease A family member 1, pancreatic
ROBO1	roundabout guidance receptor 1
ROR1	receptor tyrosine kinase like orphan receptor 1
RORA	RAR related orphan receptor A
RPS12	ribosomal protein S12
RUVBL1	RuvB like AAA ATPase 1
RUVBL2	RuvB like AAA ATPase 2
SBDS	SBDS ribosome maturation factor
SCUBE3	signal peptide, CUB domain and EGF like domain containing 3
SDC1	syndecan 1

SEMA5A	semaphorin 5A
SERPINE1	serpin family E member 1
SERPINH1	serpin family H member 1
SFRP4	secreted frizzled related protein 4
SHC1	SHC adaptor protein 1
SMAD4	SMAD family member 4
SMIM6	small integral membrane protein 6
SMOC1	SPARC related modular calcium binding 1
SNX3	sorting nexin 3
SORBS2	sorbin and SH3 domain containing 2
SORT1	sortilin 1
SPIN1	spindlin 1
SPNS1	SPNS lysolipid transporter 1, lysophospholipid
SPOCK2	SPARC (osteonectin), cwcv and kazal like domains proteoglycan 2
SPP1	secreted phosphoprotein 1
SRC	SRC proto-oncogene, non-receptor tyrosine kinase
SRPX2	sushi repeat containing protein X-linked 2
STC1	stanniocalcin 1
STRN	striatin
SULF1	sulfatase 1
SULF2	sulfatase 2
SYK	spleen associated tyrosine kinase
TAX1BP3	Tax1 binding protein 3
TBL1XR1	TBL1X/Y related 1
TF	transferrin
TFRC	transferrin receptor
TGFB1	transforming growth factor beta 1
TGFB1I1	transforming growth factor beta 1 induced transcript 1
TGFB2	transforming growth factor beta 2
TGFB3	transforming growth factor beta 3
TGFBI	transforming growth factor beta induced
TGFBR2	transforming growth factor beta receptor 2
THBS3	thrombospondin 3
THY1	Thy-1 cell surface antigen
TMEM119	transmembrane protein 119
TMEM198	transmembrane protein 198
TNFRSF11B	TNF receptor superfamily member 11b
TNFRSF12A	TNF receptor superfamily member 12A
TNXB	tenascin XB
TPP1	tripeptidyl peptidase 1
TSPAN14	tetraspanin 14
TWSG1	twisted gastrulation BMP signaling modulator 1
TYMP	thymidine phosphorylase

UBE2O	ubiquitin conjugating enzyme E2 O
UNC5B	unc-5 netrin receptor B
USP15	ubiquitin specific peptidase 15
VAV2	vav guanine nucleotide exchange factor 2
VCAM1	vascular cell adhesion molecule 1
VCP	valosin containing protein
VEGFA	vascular endothelial growth factor A
VEGFC	vascular endothelial growth factor C
VPS35	VPS35 retromer complex component
VTN	vitronectin
VWF	von Willebrand factor
WASF2	WASP family member 2
WDR12	WD repeat domain 12
WNK1	WNK lysine deficient protein kinase 1
WNT5A	Wnt family member 5A
WNT5B	Wnt family member 5B
YAP1	Yes1 associated transcriptional regulator
YWHAZ	tyrosine 3-monooxygenase/tryptophan 5-monooxygenase activation protein zeta

Supplementary Table S3: List of cytokines included in the human bone metabolism multiplex array (n=31)

AREG	Amphiregulin
BMP4	Bone morphogenetic protein 4
CCL2	C-C motif chemokine 2
CCL3	CC motif chemokine ligand 3
CDH3	P-cadherin
CDH5	VE-Cadherin
CSF1	Colony stimulating factor 1
CXCL12	Stromal cell-derived factor 1
CXCL8	Interleukin 8
FGF1	Fibroblast growth factor 1
FGF2	Fibroblast growth factor 2
GDF2	Growth differentiation factor 2
GPNMB	Glycoprotein nonmetastatic melanoma protein B (osteoactivin)
ICAM1	Intercellular Adhesion Molecule 1
IGF1	Insulin-like growth factor-I
IL11	Interleukin 11
IL17A	Interleukin 17A
IL1A	Interleukin 1 alpha
IL1B	Interleukin 1 beta
IL6	Interleukin 6
INHBA	Inhibin, beta A
MMP13	Matrix metallopeptidase 13
MMP2	Matrix metallopeptidase 2
MMP9	Matrix metallopeptidase 9
SELE	E-Selectin
SHH	Sonic Hedgehog N-Terminal
TGFB1	Transforming growth factor beta 1
TGFB2	Transforming growth factor beta 2
TNF	Tumor necrosis factor
TNFRSF11A	TNF receptor superfamily member 1A (RANK)
VCAM1	Vascular cell adhesion protein 1

Supplementary Table S4: In vivo studies of CM for bone regeneration

Study	Model	Cells	Carrier	Groups	Result
Implantation					
Osugi et al. (2)	rat calvaria	hMSC (Lonza), CDM	Agarose gel	CM<br>SFM<br>PBS<br>hMSC	4 w, CM: 49.5 $\pm$ 2.7%<br>hMSC: 40.9 $\pm$ 5.0%<br>8 w, CM: 64.4 $\pm$ 19.7%<br>MSC: 51.0 $\pm$ 3.7%, **p>0.05**
Linero et al. (3)	rabbit mandible	hASC, FBS	Plasma gel (PG)	ASC<br>CM<br>PG	45 d, PG 32%, ASC-PG 62%, CM-PG 75%,<br>ASC vs CM, **p>0.05**
Wang et al. (4)	rat calvaria	hUCSC, FBS	Hyaluronic gel	rMSC + CM<br>rMSC + PBS	BV/TV 4 w, CM: 0.04, PBS: 0.02<br>8 w, CM: 0.07, PBS: 0.04, **p<0.05**
Katagiri et al. (5)	rat calvaria	hMSC (Lonza), CDM	Atelo-Col. sponge - soaked	IGF-VEGF-TGFβ<br>CM<br>PBS	2 w, CM: 74.94 ± 19.11%<br>IGF-VEGF-TGF: 73.15 ± 11.95%, p>0.05
Katagiri et al. (6)	rat calvaria	hMSC (Lonza), CDM	Atelo-Col. sponge - soaked	CM<br>PBS	2 w, CM: 81.5 $\pm$ 2.7%<br>PBS: 60.63 $\pm$ 5.8%, p<0.05<br>4 w, CM: 93.07 $\pm$ 6.6%<br>PBS: 84.04 $\pm$ 4.9%, **p<0.05**
Katagiri et al. (7)	rat calvaria	hMSC (Lonza), CDM	Atelo-Col. sponge - soaked	CM<br>CM + anti-VEGF<br>PBS	2 w, CM: 72.3 ± 17.1%<br>PBS: 30.9 ± 6.2%,<br>CM + anti-VEGF: 33.1 ± 12.4%, **p<0.05**
Kawai et al. (8)	rat perio.	hMSC (Lonza), CDM	Atelo-Col. sponge - soaked	CM<br>PBS	2 w, 4 w, Qual
Hiraki et al. (9)	mice calvaria	SHED, FBS	Atelo-Col. sponge - soaked	SFM<br>SHED<br>SHED-CM	4 w, CM: 1.717 mm^3,<br>SHED: 0.801 mm^3<br>SFM: 0.756 mm^3, **p<0.05**<br>8 w, CM: 9.026 mm^3<br>SHED: 5.152 mm^3<br>SFM: 1.722 mm^3, **p<0.05**
Inukai et al. (10)	dog perio.	hMSC (Lonza), CDM	Atelo-Col. sponge - soaked	CM<br>PBS	4 w, CM: 4.89 ± 1.08 mm^2<br>PBS: approx. 2.3 mm^2, **p<0.05**
Nagata et al. (11)	rat perio.	hPDLSC, FBS	Col. sponge + fibrin gel	PDLSC-CM*<br>Fib-CM<br>SFM<br>*low, med, high conc.	4 w, PDL-CM best, conc. dependent
Tsuchiya et al. (12)	rat calvaria	rBMSC, FBS	PLGA mem +/- hydrophilic	CM-PLGA<br>CM-PLGA-H<br>PBS-PLGA<br>PBS-PLGA-H	4 w, CM-PLGA: 14.5%,<br>CM-PLGA-H: 22.7%, **p<0.05**<br>PBS-PLGA: 8.1%<br>PBS-PLGA-H: 9.5%<br>8 w, CM-PLGA: 24.1%,<br>CM-PLGA-H: 26.9%, **p<0.05**<br>PBS-PLGA: 15.8%<br>PBS-PLGA-H: 21.6%

Sanchooli et al. (13)	rat calvaria (hypo-thyroid)	rASC, FBS	HA (BioOss) + Col gel	CM<br>ASC	4 w, CM = ASC > scaffold<br>8 w, CM > ASC > scaffold, **p<0.05**
Wang et al. (14)	rat (diabetic) femur	hMSC, FBS	Gel sponge - soaked	CM-GS<br>SFM-GS	8 w, CM: 6.5 mm^3<br>SFM: 1.5 mm^3, **p<0.05**
Qiu et al. (15)	rat perio.	hGSC<br>hPDLSC<br>hGF, FBS	Col. Mem. (Bio-Gide) – soaked for 12 h	SFM<br>GSC-CM<br>PDL-CM<br>GF-CM<br>PBS	4 w, PDL: 48%, **p<0.05**<br>GSC: 45%, **p<0.05**<br>PDL vs GSC, **p>0.05**<br>GF: 28%<br>SFM: 25%<br>PBS: 18%
Ogisu et al. (16)	mice calvaria	hMSC,<br>hPDLF<br>FBS +/- stretch	Col. sponge	SFM<br>PDLF-CM<br>MSC-CM<br>+/- stretch	4 w, MSC-S: 45%, **p<0.05**<br>PDLF-S: 42%, **p<0.05**<br>MSC: 20%<br>PDLF: 25%<br>SFM: 5%
Diomede et al. (17)	rat calvaria	hPDLSC, FBS	Col. Mem. (Evolution)	PBS<br>PDLSC<br>PDL-CM<br>PDLSC + CM	Qual.
Diomede et al. (18)	rat calvaria	hGSC, FBS	PLA scaffold	PBS<br>GSC<br>GSC-CM<br>GSC + CM	Qual.
Pranskunas et al. (19)	rabbit calvaria	raPOSC, FBS	HA granules (Cerabone)	POSC-CM<br>POSC-osteo-CM	6 w, POSC = osteo (50%) > control (35%), **p<0.05**<br>12 w, Osteo (60%) > POSC (55%) > control (40%), **p<0.05**
Injection					
Ogata et al. (20)	rat maxilla (BRONJ)	hMSC (Lonza), CDM	I.v.	CM<br>SFM	CM > SFM, **p<0.05**
Xu et al. (21)	rat tibia DO	hFMSC, FBS	Local	CM<br>SFM<br>PBS	CM > SFM, PBS, **p<0.05**
Fujio et al. (22)	rat DO	hDPC, FBS	Local (multiple)	CM<br>CM-hypoxia<br>SFM	CM-hypo (42%) > CM (35%) > SFM (31%)<br>CM-hypo vs SFM, **p<0.05**

hMSC, human mesenchymal stromal cells (MSC), hASC, human adipose stromal cells, hUCSC, human umbilical cord stem cells, hPDLSC, human periodontal ligament stem cells, SHED, stem cells from exfoliated deciduous teeth, hGSC, human gingival stem cells, hDPC, human dental pulp stem cells, rBMSC, rat bone marrow MSC, rASC, rat adipose MSC, raPOSC, rabbit peiosteal stem cells, CDM, chemically defined medium, FBS, fetal bovine serum, SFM, serum-free media, PBS, phosphate buffered saline, CM, conditioned media, qual, qualitative, I.v., intravenous, DO, distraction osteogenesis, BRONJ, bisphosphonate-related osteonecrosis of the jaw, w, weeks.

Supplementary Figures

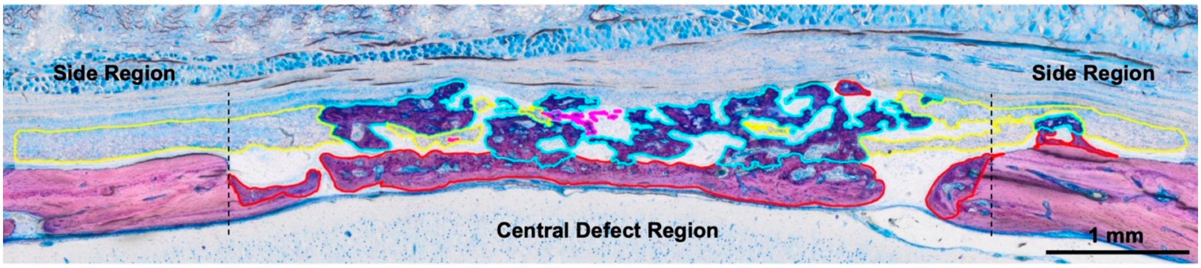

Supplementary figure S1: Histomorphometry segmentation. Representative histological section showing the different regions of interest (ROIs; central and side) and the different tissues analyzed: new bone (red), hybrid bone (cyan), mineralized fibres (pink), residual pristine membrane (yellow) and soft tissues (white). Scale bar 1 mm.

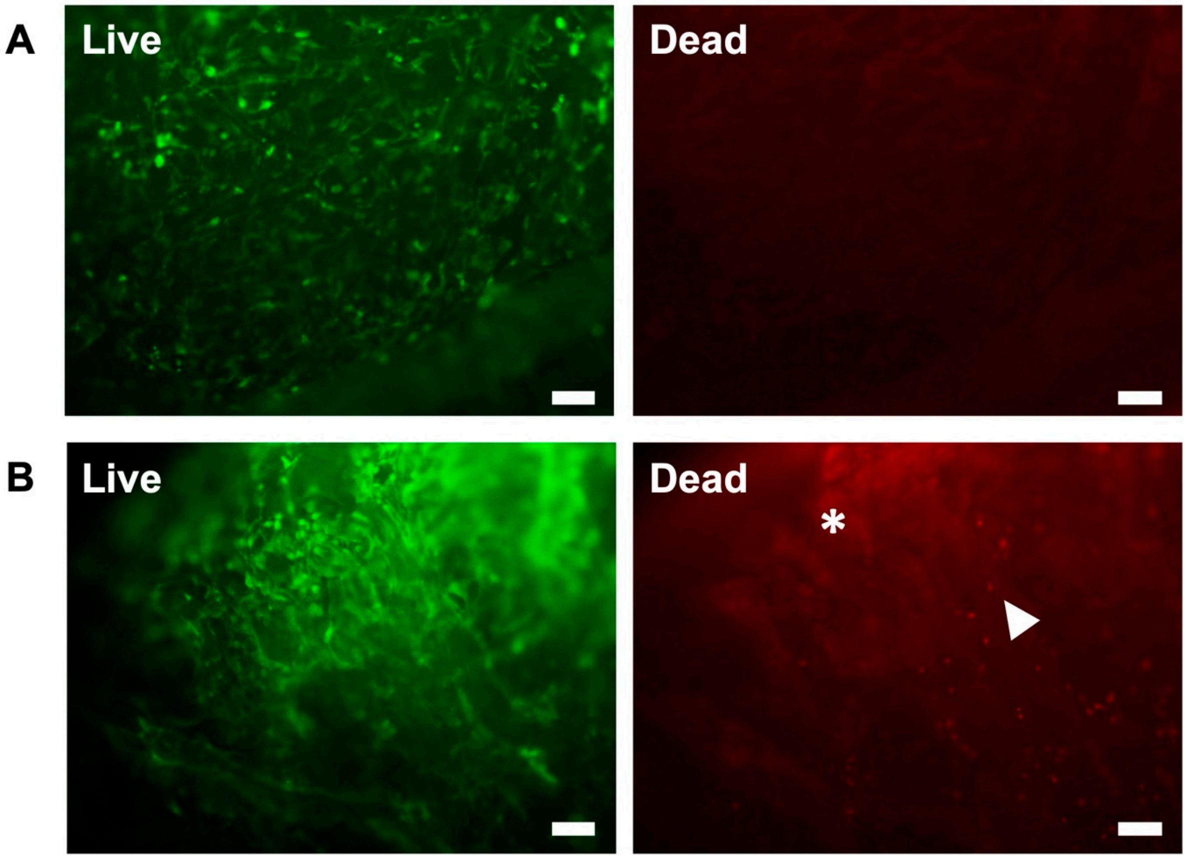

Supplementary figure S2: Cell viability on MEM. Representative images of live/dead assay of rMSC seeded on MEM for 24 hours (A) and just prior to *in vivo* implantation (B). Arrow points to dead cells and * indicates autofluorescence from the MEM. Scale bars 100 µm.

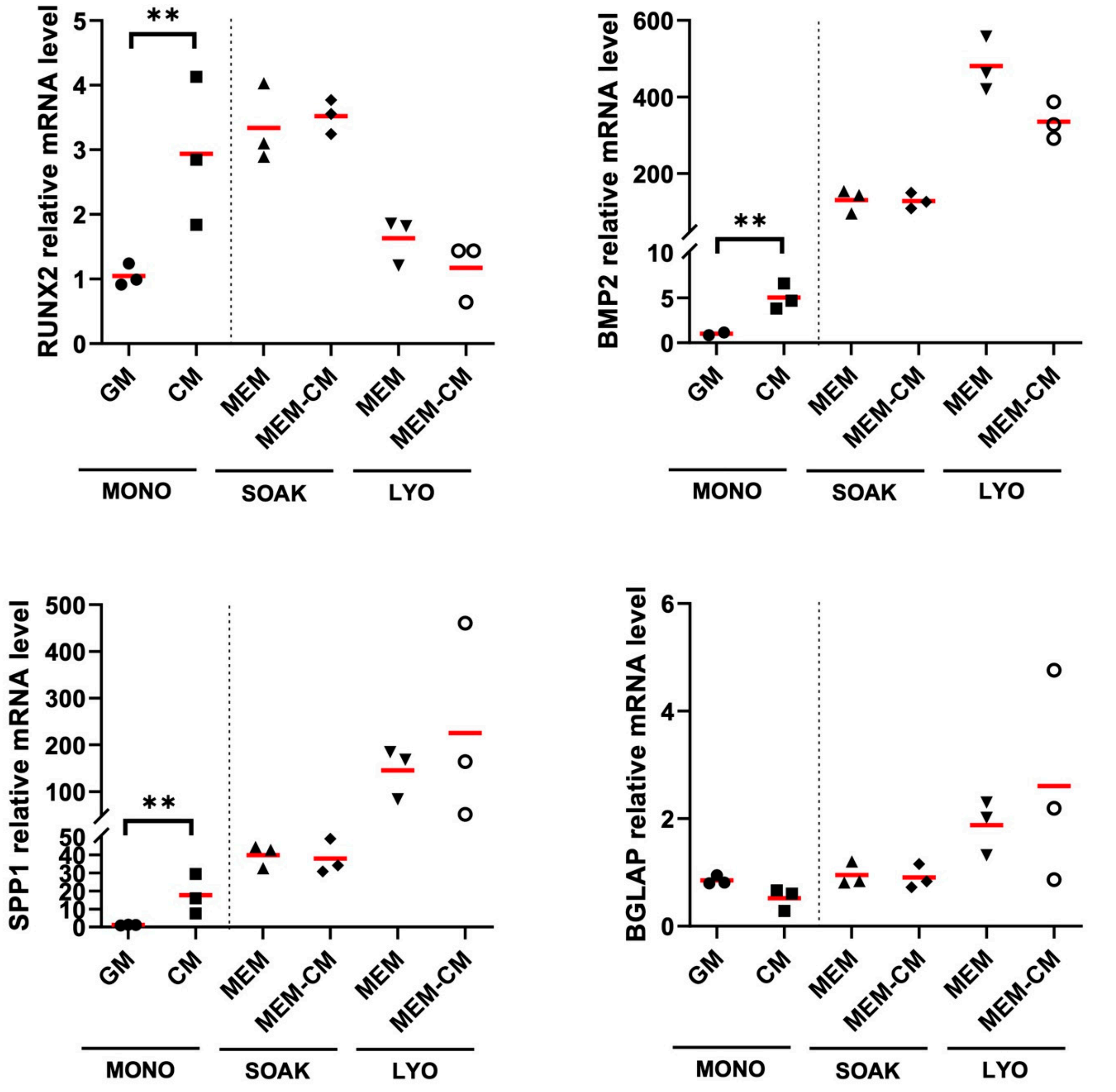

Supplementary figure S3: Gene expression assay. Relative mRNA expression of osteogenic gene markers in human BMSC after 48 hours. Data represent means (n = 3 experimental replicates); ** $p < 0.01$. BMSC were cultured as monolayers and exposed to CM or seeded on membranes (MEM; native or functionalized). Vertical dotted line indicates a distinction between the monolayer and MEM culture setups. Upregulation of genes on native MEM (even without functionalization) precluded statistical analysis. MONO, monolayer culture; GM, growth media; CM, conditioned media; MEM, collagen membrane; MEM-CM, CM functionalized MEM; SOAK, MEM soaked in CM; LYO, MEM lyophilized with CM; RUNX2, runt-related transcription factor 2; BMP2, bone morphogenetic protein 2; BGLAP/OCN, osteocalcin; SPP1/OPN, osteopontin.

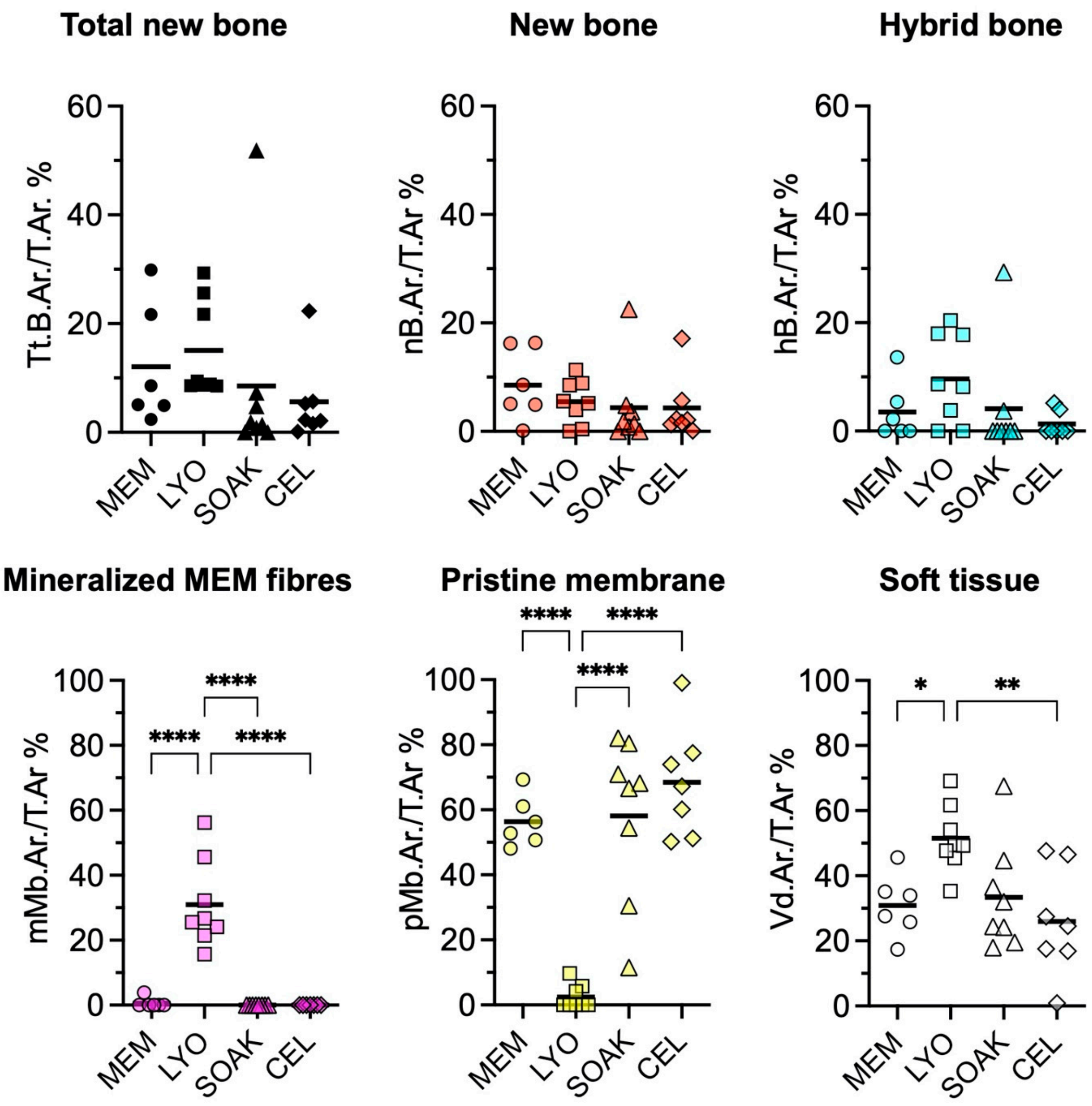

Supplementary figure S4: Histomorphometry of side regions. Quantification of histomorphometric parameters. Data represent means. * $p < 0.05$, ** $p < 0.01$, *** $p < 0.001$.

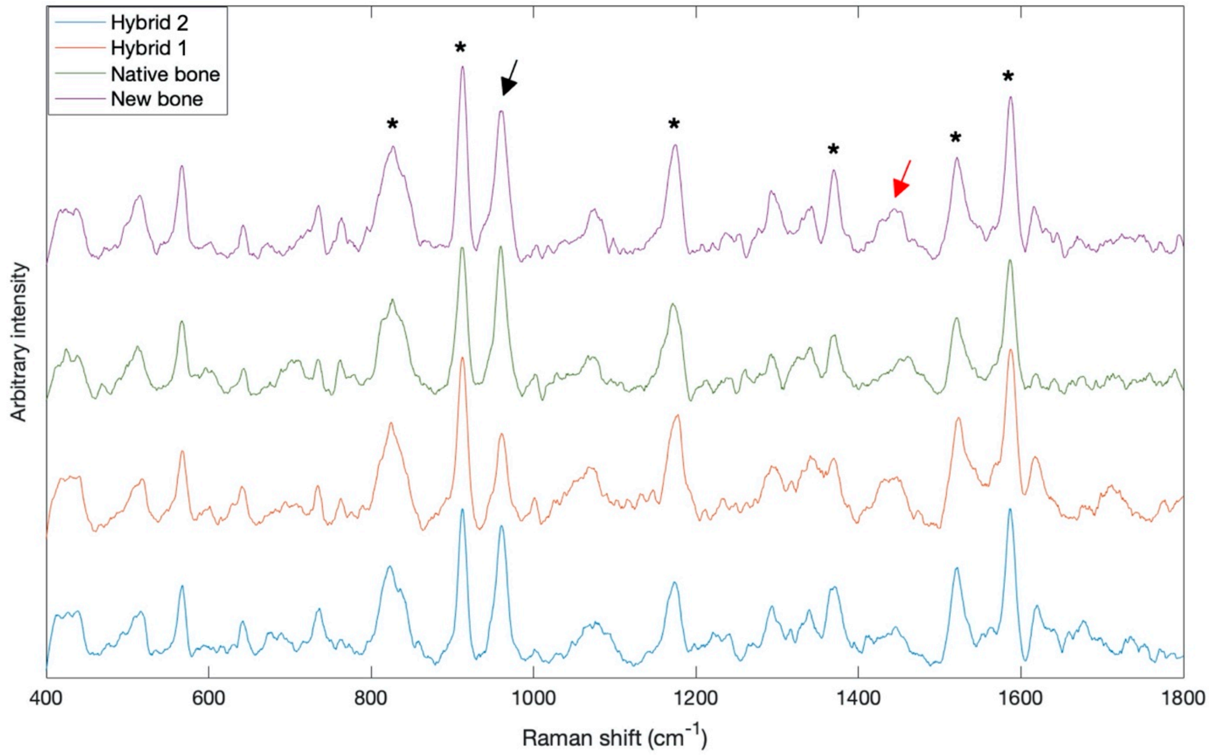

Supplementary figure S5: Raman spectroscopy. Representative Raman spectra of native bone, new bone, and hybrid bone stage 1 and stage 2. Measurements were made for two peaks of interest: phosphate ($\nu_1 PO_4^3$) peak at ~960 cm^{-1} (⟶) and CH2 wag peak at ~ 1450 cm^{-1} (⟶). * indicates peaks related to the histological staining dye (pararosaniline). All spectra were processed using a custom Matlab script for background correction and smoothing using Savitzky-Golay polynomial function in the 2nd order.

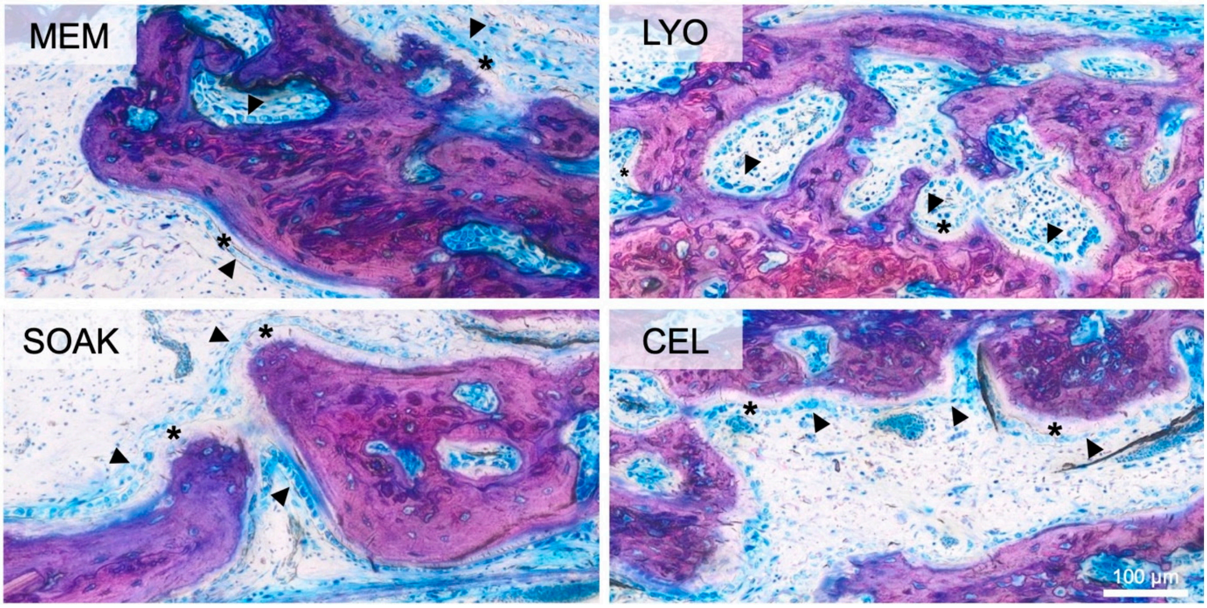

Supplementary figure S6: Histology. Representative histological images after 4 weeks showing areas of osteoblastic activity (ongoing bone formation) in the different groups as indicated by detection of characteristic osteoblast-seams (arrows) and osteoid matrix (*). Scale bar 100 µm.

Supplementary references

1. Cesaratto A, Lombardi J, Leona M. Tracking photo-degradation of triarylmethane dyes with surface-enhanced Raman spectroscopy. Journal of Raman Spectroscopy. 2016;48.
2. Osugi M, Katagiri W, Yoshimi R, Inukai T, Hibi H, Ueda M. Conditioned media from mesenchymal stem cells enhanced bone regeneration in rat calvarial bone defects. Tissue Eng Part A. 2012;18(13-14):1479-89.
3. Linero I, Chaparro O. Paracrine effect of mesenchymal stem cells derived from human adipose tissue in bone regeneration. PLoS One. 2014;9(9):e107001.
4. Wang KX, Xu LL, Rui YF, Huang S, Lin SE, Xiong JH, et al. The effects of secretion factors from umbilical cord derived mesenchymal stem cells on osteogenic differentiation of mesenchymal stem cells. PLoS One. 2015;10(3):e0120593.
5. Katagiri W, Sakaguchi K, Kawai T, Wakayama Y, Osugi M, Hibi H. A defined mix of cytokines mimics conditioned medium from cultures of bone marrow-derived mesenchymal stem cells and elicits bone regeneration. Cell Prolif. 2017;50(3).
6. Katagiri W, Osugi M, Kawai T, Ueda M. Novel cell-free regeneration of bone using stem cell-derived growth factors. Int J Oral Maxillofac Implants. 2013;28(4):1009-16.
7. Katagiri W, Kawai T, Osugi M, Sugimura-Wakayama Y, Sakaguchi K, Kojima T, et al. Angiogenesis in newly regenerated bone by secretomes of human mesenchymal stem cells. Maxillofac Plast Reconstr Surg. 2017;39(1):8.
8. Kawai T, Katagiri W, Osugi M, Sugimura Y, Hibi H, Ueda M. Secretomes from bone marrow-derived mesenchymal stromal cells enhance periodontal tissue regeneration. Cytotherapy. 2015;17(4):369-81.
9. Hiraki T, Kunimatsu R, Nakajima K, Abe T, Yamada S, Rikitake K, et al. Stem cell-derived conditioned media from human exfoliated deciduous teeth promote bone regeneration. Oral Dis. 2020;26(2):381-90.
10. Inukai T, Katagiri W, Yoshimi R, Osugi M, Kawai T, Hibi H, et al. Novel application of stem cell-derived factors for periodontal regeneration. Biochem Biophys Res Commun. 2013;430(2):763-8.
11. Nagata M, Iwasaki K, Akazawa K, Komaki M, Yokoyama N, Izumi Y, et al. Conditioned Medium from Periodontal Ligament Stem Cells Enhances Periodontal Regeneration. Tissue Eng Part A. 2017;23(9-10):367-77.
12. Tsuchiya S, Ohmori M, Hara K, Fujio M, Ikeno M, Hibi H, et al. An Experimental Study on Guided Bone Regeneration Using a Polylactide-co-glycolide Membrane-Immobilized Conditioned Medium. Int J Oral Maxillofac Implants. 2015;30(5):1175-86.
13. Sanchooli T, Norouzian M, Ardeshirylajimi A, Ghoreishi S, Abdollahifar M, Nazarian H, et al. Adipose Derived Stem Cells Conditioned Media in Combination with Bioceramic-Collagen Scaffolds Improved Calvarial Bone Healing in Hypothyroid Rats. Iranian Red Crescent Medical Journal. 2017;In Press.
14. Wang CY, Yang HB, Hsu HS, Chen LL, Tsai CC, Tsai KS, et al. Mesenchymal stem cell-conditioned medium facilitates angiogenesis and fracture healing in diabetic rats. J Tissue Eng Regen Med. 2012;6(7):559-69.
15. Qiu J, Wang X, Zhou H, Zhang C, Wang Y, Huang J. Enhancement of periodontal tissue regeneration by conditioned media from gingiva-derived or periodontal ligament-derived mesenchymal stem cells: a comparative study in rats. Stem Cell Res. Ther. 2020.
16. Ogisu K, Fujio M, Tsuchiya S, Tsuboi M, Qi C, Toyama N, et al. Conditioned media from mesenchymal stromal cells and periodontal ligament fibroblasts under cyclic stretch stimulation promote bone healing in mouse calvarial defects. Cytotherapy. 2020;22(10):543-51.
17. Diomede F, D'Aurora M, Gugliandolo A, Merciaro I, Orsini T, Gatta V, et al. Biofunctionalized Scaffold in Bone Tissue Repair. Int J Mol Sci. 2018;19(4).

18. Diomede F, Gugliandolo A, Scionti D, Merciaro I, Cavalcanti MF, Mazzon E. Biotherapeutic effect of gingival stem cells conditioned medium in bone tissue restoration. Int J Mol Sci. 2018;8:315-7.
19. Pranskunas M, Simoliunas E, Alksne M, Martin V, Gomes PS, Puisys A, et al. Assessment of the Bone Healing Process Mediated by Periosteum-Derived Mesenchymal Stem Cells' Secretome and a Xenogenic Bioceramic-An In Vivo Study in the Rabbit Critical Size Calvarial Defect Model. Materials (Basel). 2021;14(13).
20. Ogata K, Katagiri W, Osugi M, Kawai T, Sugimura Y, Hibi H, et al. Evaluation of the therapeutic effects of conditioned media from mesenchymal stem cells in a rat bisphosphonate-related osteonecrosis of the jaw-like model. Bone. 2015;74:95-105.
21. Xu J, Wang B, Sun Y, Wu T, Liu Y, Zhang J, et al. Human fetal mesenchymal stem cell secretome enhances bone consolidation in distraction osteogenesis. Stem Cell Res Ther. 2016;7(1):134.
22. Fujio M, Xing Z, Sharabi N, Xue Y, Yamamoto A, Hibi H, et al. Conditioned media from hypoxic-cultured human dental pulp cells promotes bone healing during distraction osteogenesis. J Tissue Eng Regen Med. 2017;11(7):2116-26.
